# Cenozoic Methane-Seep Faunas of the Caribbean Region

**DOI:** 10.1371/journal.pone.0140788

**Published:** 2015-10-15

**Authors:** Steffen Kiel, Bent T. Hansen

**Affiliations:** 1 Georg-August-Universität Göttingen, Geoscience Center, Geobiology Group, Goldschmidtstr. 3, 37077, Göttingen, Germany; 2 Naturhistoriska riksmuseet, Department of Palaeobiology, Box 500 07, 104 05, Stockholm, Sweden; 3 Georg-August-Universität Göttingen, Geoscience Center, Department of Isotope Geology, Goldschmidtstr. 3, 37077, Göttingen, Germany; Union College, UNITED STATES

## Abstract

We report new examples of Cenozoic cold-seep communities from Colombia, Cuba, the Dominican Republic, Trinidad, and Venezuela, and attempt to improve the stratigraphic dating of Cenozoic Caribbean seep communities using strontium isotope stratigraphy. Two seep faunas are distinguished in Barbados: the late Eocene mudstone-hosted ‘Joes River fauna’ consists mainly of large lucinid bivalves and tall abyssochrysoid gastropods, and the early Miocene carbonate-hosted ‘Bath Cliffs fauna’ containing the vesicomyid *Pleurophopsis*, the mytilid *Bathymodiolus* and small gastropods. Two new Oligocene seep communities from the Sinú River basin in Colombia consist of lucinid bivalves including *Elongatolucina*, thyasirid and solemyid bivalves, and *Pleurophopsis*. A new early Miocene seep community from Cuba includes *Pleurophopsis* and the large lucinid *Meganodontia*. Strontium isotope stratigraphy suggests an Eocene age for the Cuban Elmira asphalt mine seep community, making it the oldest in the Caribbean region. A new basal Pliocene seep fauna from the Dominican Republic is characterized by the large lucinid *Anodontia* (*Pegophysema*). In Trinidad we distinguish two types of seep faunas: the mudstone-hosted Godineau River fauna consisting mainly of lucinid bivalves, and the limestone-hosted Freeman’s Bay fauna consisting chiefly of *Pleurophopsis*, *Bathymodiolus*, and small gastropods; they are all dated as late Miocene. Four new seep communities of Oligocene to Miocene age are reported from Venezuela. They consist mainly of large globular lucinid bivalves including *Meganodontia*, and moderately sized vesicomyid bivalves. After the late Miocene many large and typical ‘Cenozoic’ lucinid genera disappeared from the Caribbean seeps and are today known only from the central Indo-Pacific Ocean. We speculate that the increasingly oligotrophic conditions in the Caribbean Sea after the closure of the Isthmus of Panama in the Pliocene may have been unfavorable for such large lucinids because they are only facultative chemosymbiotic and need to derive a significant proportion of their nutrition from suspended organic matter.

## Introduction

Methane seeps on the deep-sea floor harbor dense faunal communities whose dominant members rely on chemosynthetic bacteria for nutrition [1, 2]. First discovered in the Gulf of Mexico in the 1980s, they are now recognized at virtually all continental margins worldwide [3, 4]. Although the rise of the modern, mollusk-dominated vent and seep fauna began during the Cretaceous, the most common taxa at present-day vents and seeps originated in the early Cenozoic [5–9]. Biogeographically the Cenozoic fossil record of methane seeps is strongly skewed toward the active continental margins of the Pacific Ocean [10–13]; fossil occurrences in the Atlantic realm are restricted to the Caribbean region [14, 15] and the Mediterranean basin [16, 17]. The fossil record of the Caribbean region is of particular interest in this context because this area has long served as a gateway for faunal exchange between the Atlantic and Pacific oceans [18, 19].

Unusual faunal assemblages have been reported for a long time from the Caribbean region, including examples from Trinidad that were dominated by the enigmatic bivalve *Pleurophopsis* Van Winkle, 1919 [20, 21], from Cuba, which was considered as a mix of marine and freshwater taxa [22], from Colombia, where the ‘*Pleurophopsis* fauna’ was reported from the Oligocene of the Sinú River basin [23], and a fauna from the Joes River area in Barbados, which “appears to be a specialized one, perhaps requiring an unusual environment for its existence” [24]. These and other, similar faunas were subsequently identified as ancient methane seep faunas [14, 15, 25], but the still poor age determinations and taxonomic identifications of these faunas have so far prevented a rigorous analysis of the evolution of seep faunas from the Caribbean/Gulf of Mexico region, as well as their role in the biogeographic evolution of the seep fauna in general.

**Fig 1:**
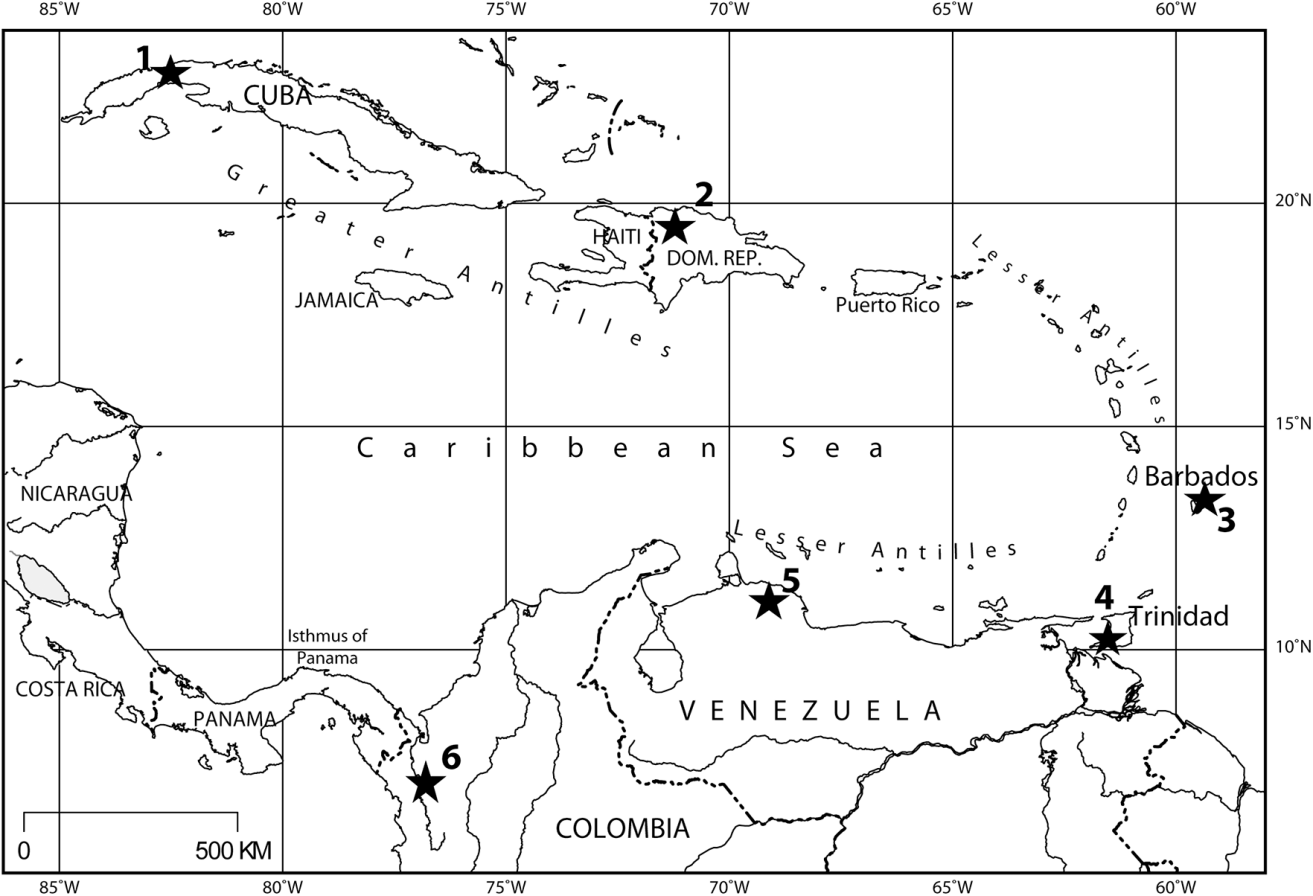
Locality map showing the seep faunas reported herein. 1: Cantera Portugalete and Elmira asphalt mine, Cuba; 2: Cañada de Zamba, Dominican Republic; 3: Bath Cliffs and Joes River, Barbados; 4: Bronte Estate, Freeman’s Bay, Godineau River, and Jordan Hill, Trinidad; 5: Buenavista de Maicillal, Caujarao, Corro Colorado, La Piedra and Puerto Escondido, Venezuela; 6: Mata Cana and Sta. Clara, Colombia.

Here we report new seep faunas from Colombia, Cuba, the Dominican Republic, Trinidad, and Venezuela, and our attempts to improve the dating of some of the known faunas from Barbados, Cuba, Trinidad, and Venezuela (Fig 1), based on strontium isotope stratigraphy. A comprehensive taxonomic account on the mollusk species of the Caribbean Cenozoic seep faunas is beyond the scope of the present paper and will be published separately.

Sr isotope stratigraphy is based on the observation that (i) marine carbonates record the ^87^Sr/^86^Sr-ratio of the seawater in which they formed, and (ii) those ratios have changed through Earth’s history. Thousands of measurements of samples with known biostratigraphic ages have been compiled into a Phanerozoic Sr-isotope curve that can be used, within certain limits, to infer the age of a marine carbonate sample based on its ^87^Sr/^86^Sr-ratio [26]. We have recently tested whether this approach can be applied to fossil methane-seep carbonates [27]. Methane seeps pose the potential risk that the seeping fluid carries Sr with a ^87^Sr/^86^Sr-ratio quite different from that of ambient seawater [28], which could corrupt the utility of the Sr isotope signature of the seep carbonate for stratigraphic purposes. However, this does not seem to the case as most tested Recent seep carbonates carry the marine Sr isotope signature [29, 30], and our tests with a wide range of Phanerozoic seep carbonates indicated that diagenetic alteration, not initial contamination, is the main issue in this approach [27].

## Material and Methods

The new faunas reported here are from the collection of the Paleontological Research Institution (PRI) in Ithaca, USA, and the Naturhistorisches Museum Basel (NMB), Switzerland. All taxonomic identifications and assignments presented here were made by SK, except when noted otherwise. Additional material for Sr isotope stratigraphy and thin sectioning was provided by the Smithsonian Natural History Museum (USNM) in Washington, DC, USA, for the Palmar-Molinera-road site in Colombia, and the Elmira asphalt mine site in Cuba [15]. The fossil occurrences are identified as ancient methane seeps based on (i) their faunal content, typically large lucinid, vesicomyid, and mytilid bivalves, (ii) the carbon isotope values (δ^13^C) of the associated carbonate, which tend to be very negative, and (iii) distinct carbonate fabrics of the associated carbonate (when available). The oxygen isotope signature (δ^18^O) of the associated carbonate is used as an indicator for the degree of diagenetic alteration of the carbonate, which is used to assess the reliability of the Sr isotope signature [27, 31]. No permits were required for the described study, which complied with all relevant regulations.

Strontium isotopic compositions of representative samples were analyzed on unspiked samples. The samples were weighted into Teflon vials and dissolved in a mixture of 5 ml HF and HNO_3_ (3:2) with a PicoTrace digestion system. This form of digestion may imply that tiny amounts of detrital and/or meteoric material are dissolved as well, and therefore the obtained ratios represent maximum ages. The solutions were processed by standard cation-exchange techniques for purification of the Sr fractions. Sr was loaded with 0.5N H_3_PO_4_ on pre-conditioned double Re filaments. Measurements of isotopic ratios were performed on a ThermoFinnigan Triton mass spectrometer in static mode (GZG Göttingen, Dept. Isotope Geology). The mean ^87^Sr/^86^Sr ratio obtained for the Sr standard SRM NBS987 during the period of analytical work was 0.710272 ± 0,000039 (n = 8, 2σ). All Sr isotopic ratios were normalized to an ^86^Sr/^88^Sr ratio of 0.1194 over the course of this study. Total procedure blanks were consistently below 150 pg. ^87^Sr/^86^Sr ratios given as “^87^Sr/^86^Sr [corr.]” in Table 1 are corrected for blank and mass fractionation, and after these corrections the ^87^Sr/^86^Sr ratios were adjusted to 0.710248 for the NBS987 which is the normalization ratio for the LOWESS curve and look-up table version 5.0 [26]. All ^87^Sr/^86^Sr ratios are reported with their 2 σ internal precision. For further details see [32]. Samples for Sr isotope analyses were either taken from the shells of mollusks, or from matrix micrites or rim cements of the seep carbonates that contain the mollusks. The latter carbonate phases often preserve an unaltered Sr isotope signal [27] and the samples were checked for diagenetic alteration using their oxygen isotope signature (Table 1).

**Table 1:** Strontium isotope data and derived ages.

[Sample number], origin of sample	^87^Sr/^86^Sr [corr.]	2se	δ^13^C (PDB)[‰]	δ^18^O (PDB)[‰]	Age [numerical, stage]
[Sr-1] Barbados, NMB loc. 10147, G17898, peloidal micrite	0.708306	0.000007	-15.3	1.4	22.05 (+0.8/-0.55), lower Aquitanian
[Sr-1] Barbados, NMB loc. 10147, G17898, peloidal micrite	0.708335	0.000012	-15.3	1.7	21.6 (+0.7/-0.7), middle Aquitanian
[Sr-3] Barbados, NMB loc. 10147, G17899, micrite	0.708509	0.000010	-14.3	-1.2	18.75, lower Burdigalian
[Sr-4] Barbados, gastropod shell, NMB loc. 10039, H20157	0.707745	0.000017	2.6	1.4	37.6 (+n.a./-3), basal Priabonian
[Sr-4] Barbados, gastropod shell, NMB loc. 10039, H20157	0.707750	0.000010	2.1	1.3	37.2 (+n.a./-0.9), basal Priabonian
[Sr-6] Colombia, Mata Cana, micrite	0.707535	0.000007	-36.6	-1.9	* below Cenozoic Sr isotope curve
[Sr-7] Colombia, Mata Cana, micrite	0.707493	0.000016	-32.8	-1.9	* below Cenozoic Sr isotope curve
[Sr-8] Colombia, Sta. Clara, micrite	0.707507	0.000006	-23.0	-5.9	* below Cenozoic Sr isotope curve
[Sr-9] Colombia, Sta. Clara, micrite	0.707556	0.000014	-24.5	-5.7	* below Cenozoic Sr isotope curve
[Sr-10] Colombia, Palmar-Molinera, rim cement, USNM 558830	0.708663	0.000005	-52.5	-5.9	16.85 uppermost Burdigalian
[Sr-10] Colombia, Palmar-Molinera, rim cement, USNM 558830	0.708688	0.000006	-50.8	-5.8	16.5, uppermost Burdigalian
[Sr-12] Colombia, Palmar-Molinera, rim cement, different sample	0.708631	0.000013	-45.0	-3.8	17.3, upper Burdigalian
[Sr-13] Cuba, Elmira Asphalt mine, micrite from concretion	0.707731	0.000007	-28.2	-0.3	39.0 (+1/-4), Bartonian
[Sr-14] Cuba, Elmira Asphalt mine, micrite from concretion	0.707697	0.000006	-28.7	-0.4	49.25 (+n.a./-1.65), upper Ypresian
[Sr-15] Cuba, Elmira Asphalt mine, *Unio*? *bitumen* shell	0.707705	0.000007	-2.4	-1.8	48.8 (+n.a./-1.6), upper Ypresian
[Sr-16] Cuba, Cantera Portugalete, white micrite	0.708459	0.000005	-7.0	1.7	19.4 (+0.4/-0.9), basal Burdigalian
[Sr-17] Cuba, Cantera Portugalete, micrite	0.708450	0.000005	-9.2	0.6	19.5 (+0.5/-0.6), basal Burdigalian
[Sr-18] Trinidad, Godineau River, bivalve shell	0.708855	0.000006	-16.9	-2.9	11.1, basal Tortonian
[Sr-19] Trinidad, Godenau River, micrite	0.708952	0.000007	-20.7	3.7	6.35 (+2.65/-0.35), middle Messinian
[Sr-20] Trinidad, Freeman's Bay, light micrite	0.709027	0.000011	-14.5	-1.8	* 5.3, Miocene-Pliocene boundary
[Sr-21] Trinidad, Freeman's Bay, brown micrite	0.709120	0.000007	-20.0	-2.3	* 1.3, Pleistocene
[Sr-22] Trinidad, Jordan Hill, micrite	0.708936	0.000008	-43.7	2.6	7.7 (+2/-1.3), uppermost Tortonian
[Sr-23] Trinidad, Jordan Hill, micrite	0.708918	0.000007	-29.3	0.0	8.8 (+1.2/-1.8), upper Tortonian
[Sr-24] Venezuela, Corro Colorado, micrite	0.708481	0.000014	-22.0	-2.7	19.1 (+/- 0.5), lower Burdigalian
[Sr-25] Venezuela, Corro Colorado, micrite	0.708394	0.000004	ND	ND	20.5 (+0.75/-0.7), uppermost Aquitanian
[Sr-26] Venezuela, Buena Vista de Maicillal, micrite	0.708342	0.000018	-21.4	-2.4	21.45 (+0.65/-0.7), Aquitanian
[Sr-27] Venezuela, Buena Vista de Maicillal, micrite	0.708292	0.000028	-21.8	-2	22.3 (+0.8/-0.65), basal Aquitanian
[Sr-28] Venezuela, Buena Vista de Maicillal, micrite	0.708354	0.000011	-21.3	-2.6	21.25 (+0.7/-0.75), Aquitanian
[Sr-29] Venezuela, Caujarao, micrite	0.708394	0.000033	-23.0	-4.3	20.5 (+0.75/-0.7), uppermost Aquitanian
[Sr-30] Venezuela, La Piedra, micrite	0.708971	0.000008	12.4	-5.9	6.45 (+2.6/-0.8), Messinian
[Sr-31] Venezuela, La Piedra, micrite	0.708868	0.000005	-2.1	-6.5	10.75 (+2.45/-1.35), lower Tortonian
[Sr-32] Venezuela, Puerto Escondido, micrite	0.708946	0.000006	-26.5	-3.6	7.2 (+2.1/-1.1), Tortonian/Messinian
[Sr-33] Venezuela, Puerto Escondido, micrite	0.708979	0.000008	-24.5	-1.4	6.25 (+1.25/-0.75), Messinian

* = derived ages that are considered unrealistic because the oxygen isotope signal suggests diagenetic alteration; ND = do data; n.a. = not applicable because the value was below the Cenozoic Sr isotope curve.

Samples for carbon and oxygen isotope analyses were extracted using a hand-held microdrill and carbonate powders were reacted with 100% phosphoric acid at 75°C using a Kiel III online carbonate preparation line connected to a ThermoFinnigan 252 mass spectrometer. All values are reported in per mil relative to V-PDB by assigning a δ^13^C value of +1.95‰ and a δ^18^O value of –2.20‰ to NBS19. Reproducibility was checked by replicate analysis of laboratory standards and is better than ± 0.05‰.

## Barbados

**Fig 2:**
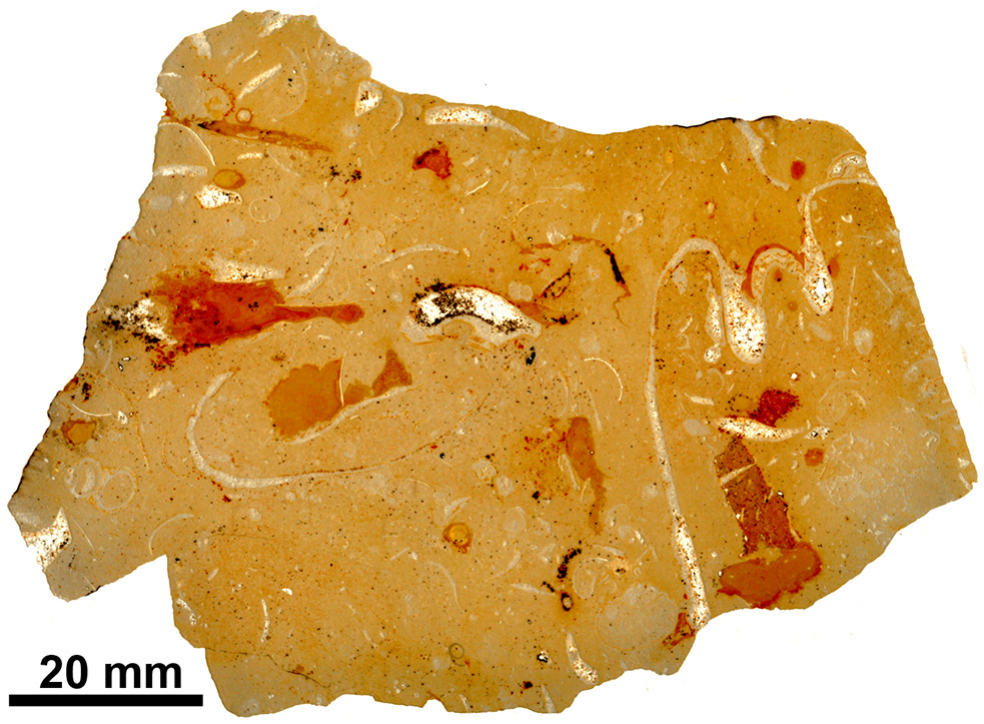
Petrography of an early Miocene seep carbonate from Barbados, NMB locality 10147. Scanned thin section, note cross section of vesicomyid bivalve on right side.

Fossil seep faunas in Barbados have been the subject of various studies, but the identity of the taxa and their stratigraphic ages are still poorly understood. An unusual mollusk fauna was reported from Eocene, oil-indurated mudstones of the Joes River Formation in Barbados [24]. Later, Harding [25] reported an early Miocene carbonate-hosted seep fauna with various bivalves and worm tubes from the nearby Bath Cliffs area, and identified the ‘Joes River’ fauna as seep related and as being of Miocene instead of Eocene age. A new geologic framework was adopted for these faunas by Gill et al. [14]: the fauna of the Joes River Formation was regarded as belonging to the diapiric mélange, and the carbonate-hosted fauna reported by [25] as belonging to the Suboceanic Fault Zone. Furthermore, they suggested that both faunas could have any geologic age between Eocene and Miocene [14]. To improve the dating of these faunas, five Sr isotope measurements were made (Table 1): three on the micritic matrix of carbonates associated with *Pleurophopsis*- and *Bathymodiolus*-like bivalves from the Oceanic Formation (samples Sr-1 to Sr-3), now the Suboceanic Fault Zone (Fig 2; Basel Museum locality 10147; from the East facing side of Bissex Hill near Cambridge, considered Middle-Early Eocene), and two on a mudstone-hosted shell of an abyssochrysoid gastropod from the Joes River Formation (samples Sr-4 and Sr-5), now diapiric mélange (Basel Museum locality 10039; western branch of Spa River, between Spa and Richmond Ridge, considered as middle Eocene).

The two samples from the gastropod (samples Sr-4 and Sr-5) indicate an early late Eocene age (basal Priabonian, 37.2 to 37.6 Ma). This is consistent with the previously suggested Eocene age [24] for the fauna associated with the Joes River Formation, which consists chiefly of large lucinid bivalves, a large bivalve of unknown affinity, the nuculanid bivalve *Nuculana senni* Kugler, Jung and Saunders, 1984, and a large abyssochrysoid gastropod now assigned to the genus *Ascheria* [24, 33]. This late Eocene mudstone-hosted association will be referred to as ‘Joes River fauna’ in the following text. The three samples from the carbonates of the ‘Oceanic Formation’ (samples Sr-1 to Sr-3) indicate an early Miocene age, the two more reliable ones indicate a lower early Miocene (Aquitanian) age, consistent with the age given by [25]. These early Miocene faunas will be referred to hereafter as the ‘Bath Cliffs fauna’.

## Colombia

One Cenozoic seep deposit has been reported from Colombia so far, called Palmar-Molinera-road locality, based on a small collection deposited in the Smithsonian Natural History Museum in Washington, DC. It consists mainly of a solemyid bivalve, two lucinid bivalves including *Elongatolucina peckmanni* Kiel, 2013 and the mussel *Bathymodiolus palmarensis* Kiel, Campbell and Gaillard, 2010 [15, 34, 35]. Here we report new petrographic data for this deposit, and two new seep faunas based on collections made by Axel A. Olsson in the 1940s, now deposited in the PRI.

### The Palmar-Molinera-road site

#### Paleoecology and stratigraphic age

**Fig 3:**
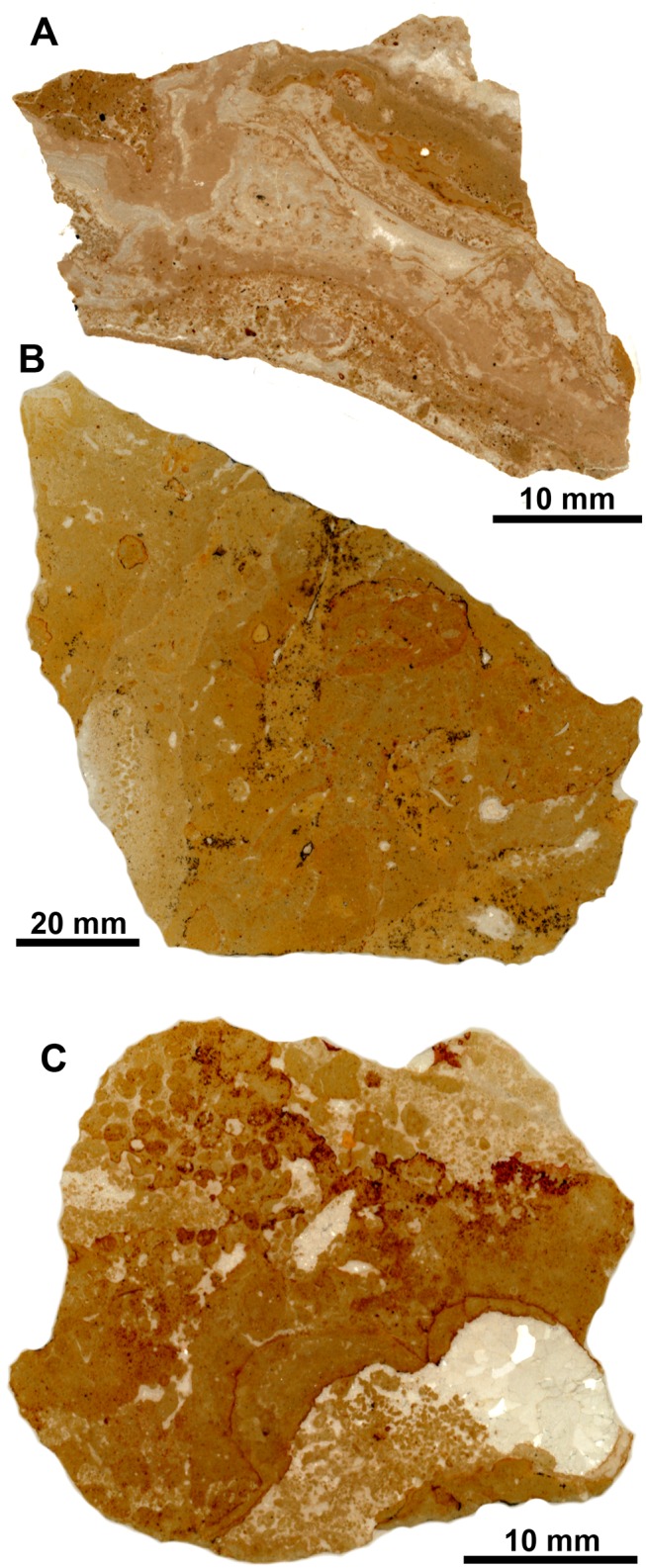
Petrography of the Oligocene seep carbonates from Colombia. Scanned thin sections. A: Palmar-Molinera Road. B. Mata Cana. C. Sta. Clara.

Thin sections made from voucher material from this locality show a micritic matrix with glauconitic grains, oval fecal pellets, detritus such as foraminiferan tests and gastropod shells, and signs of bioturbation (Fig 3A). Former voids with banded and botryoidal rim cements and filled by sparry calcite are common. In an attempt to improve the stratigraphic age of the Palmar-Molinera-road locality, three Sr isotope measurements were made from rim cements in two different samples (samples Sr-10 to Sr-12). They indicate an early Miocene age (late Burdigalian, 16.5 to 17.3 Ma) rather than an Oligocene age as indicated on the label associated with this collection.

### Mata Cana

#### Location and stratigraphic age

The labels for this collection read “Mata Cana” or “Matacona”, and “San José ls., Field along cut rd., Lower Oligocene”. Considering Olsson’s (1940) statement about the frequent occurrence of carbonate lenses with the *Pleurophopsis* fauna in the Sinú River basin mentioned above, this could possibly refer to Mata de Caña, about 20 km S of Lorica in the Department Córdoba, along the Sinú River, at 9°04’30” N, 75°49’30”W.

**Fig 4:**
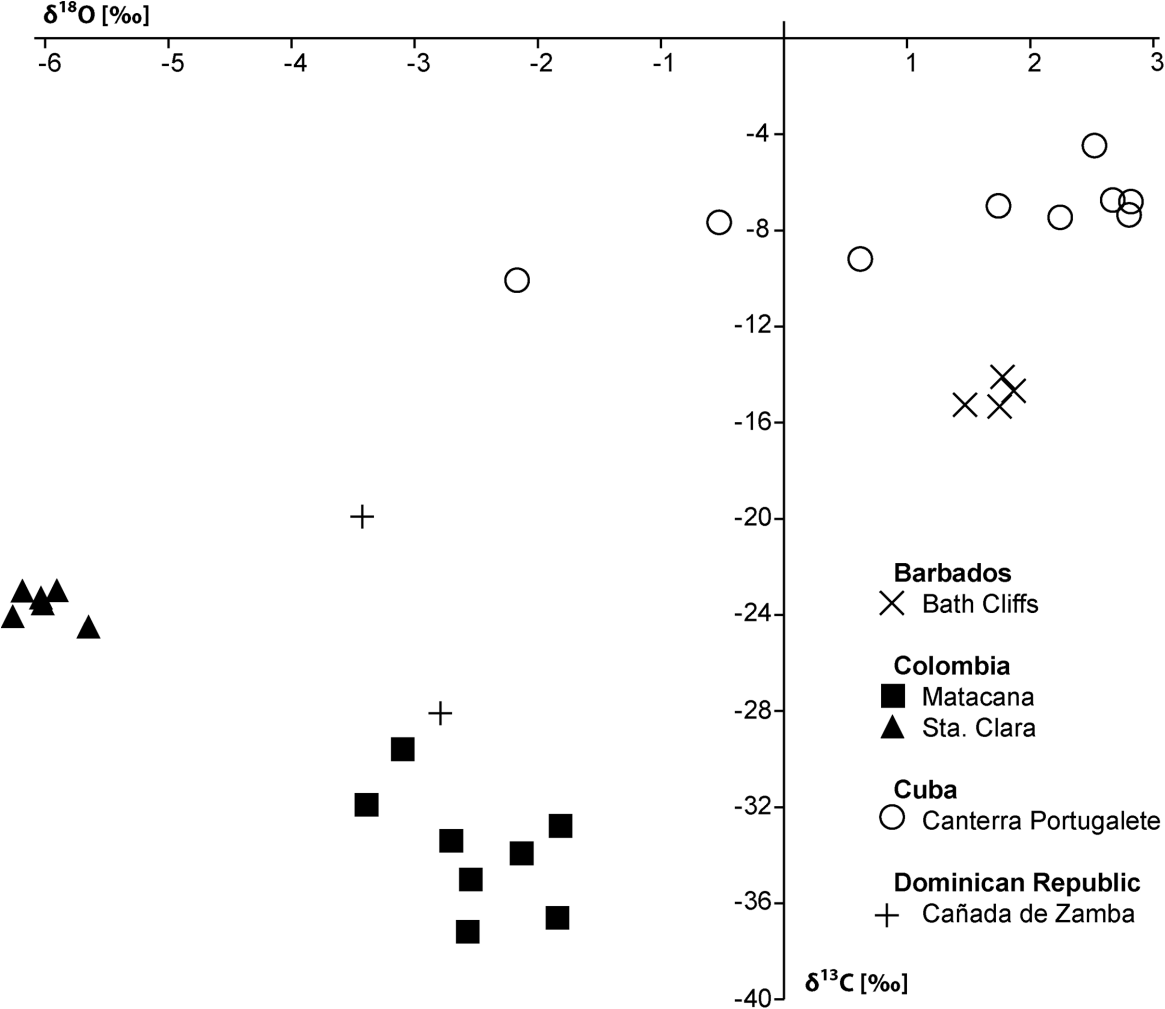
Cross-plot of stable carbon and oxygen isotope data. Barbados, Colombia, Cuba and the Dominican Republic.

The carbonate consists mostly of a peloidal micrite with microdetritus and abundant signs of bioturbation. It also contains a few small vugs that lack rim cements but are filled by sparry calcite (Fig 3B). The carbon isotope values range from –37.2 to –29.6‰ (Fig 4), indicating methane seepage [36]. The corresponding oxygen isotope values have a narrow range from –3.4 to –1.8‰ and thus indicate some diagenetic alteration; the two Sr isotope measurements (samples Sr-6 and Sr-7) fall below the Sr isotope curve for the Cenozoic (Table 1).

#### Fauna

**Fig 5:**
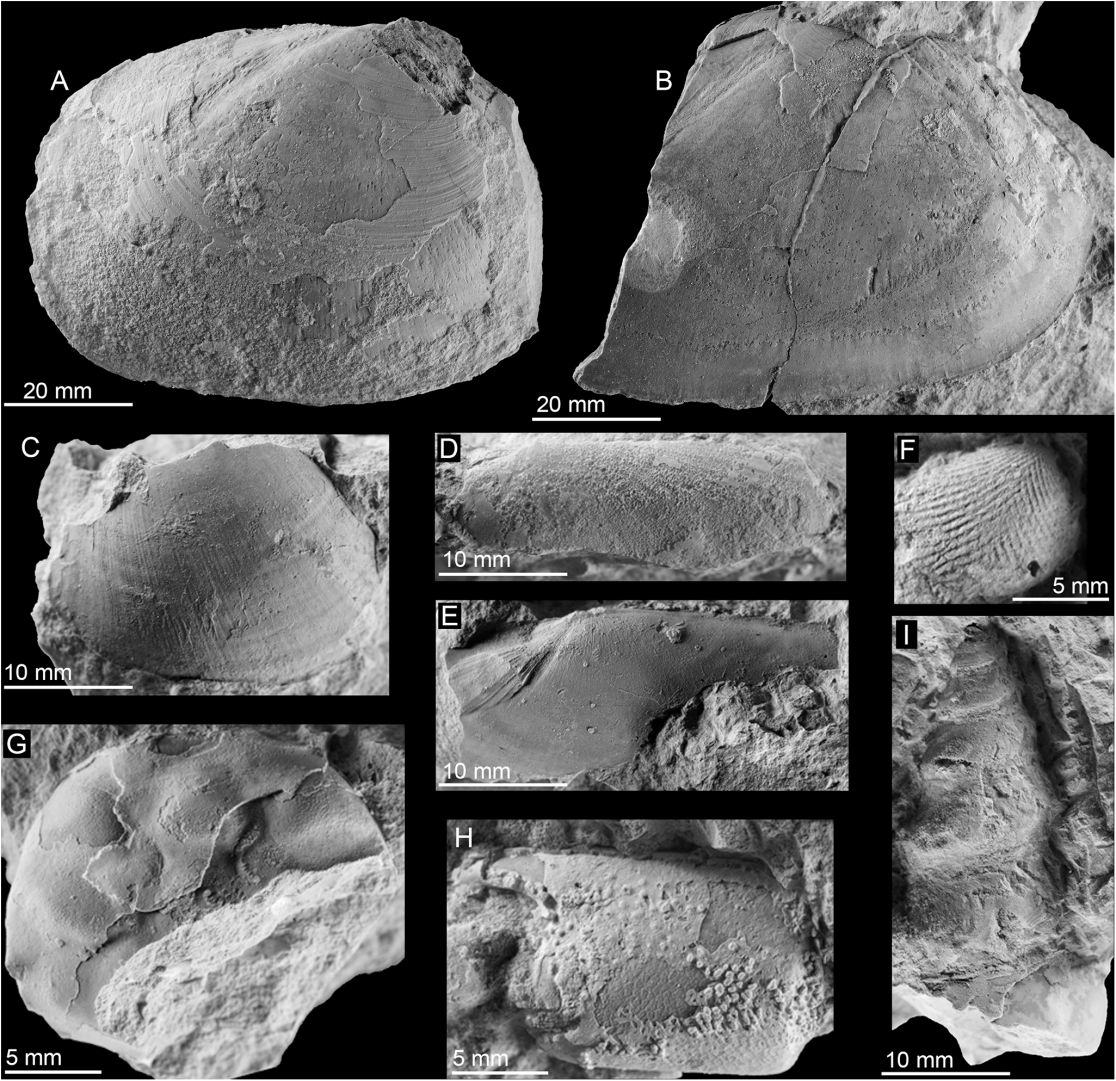
The Oligocene Mata Cana seep fauna from Colombia. A, B: The lucinid *Elongatolucina* sp. (PRI 68645, 68646). C: Oval lucinid bivalve (PRI 68647). D, E: The solemyid *Solemya* cf. *belensis* (Olsson, 1931) (PRI 68648). F: The nuculid *Truncacila* sp. (PRI 68649). G: Carapace of a ?goneplacoid crab (PRI 68650). H: Crustacean claw (PRI 68651). I: Tall neogastropod (PRI 68652).

Few fossil specimens are preserved with recrystallized shell; most are internal molds. Crustacean claws, possibly of *Callianassa* Leach, 1814, are abundant in this collection, and there is a crustacean carapace. Bivalves are also common and comprise the lucinids *Elongatolucina* Gill and Little, 2013 and a rounded-oval species with fine concentric sculpture, a very elongate solemyid resembling *Solemya belensis* Olsson, 1931 from Oligocene seep deposits in Peru [37], and the nuculid *Truncacila* Schenck, 1931; there is a high-spired gastropod, a nautiloid, and a crustacean carapace belonging to a? goneplacoid crab. The fauna is summarized in Table 2 and illustrated in Fig 5.

**Table 2:** List of taxa from Mata Cana, Colombia.

Species	Max. size (mm)	N of specimens
*Solemya* aff. *belensis* Olsson 1931	53.7	2
*Truncacila* sp.	8	2
*Elongatolucina* sp.	83.5	2
Oval lucinid	48	7
High-spired gastropod	34.5	1
Nautiloid	37	1
?Goneplacoid carapax	20	1
Crustacean claws	18	6

### Sta. Clara, loc. 303

#### Location and stratigraphic age

The label associated with this collection reads “Sta. Clara, sinú, 7225 i, flfs. ls., 303, 22-IV-40”. A screening of Axel Olsson’s field notes (Xerox copies in the USNM Cenozoic mollusk type collection) revealed no information about these potential locality numbers. But in a report on the Oligocene of western Columbia Olsson (1940, p. 250) writes that “The (middle) Oligocene beds are succeeded by shales, very similar to the Uscari and Tapaliza of Panamá and Costa Rica and at several places, particularly in the Sinú, contain limestone lenses carrying the *Pleurophopsis* fauna.” [23]. The collection includes elongate vesicomyid bivalves that were typically referred to as *Pleurophopsis* by Olsson [37] and it is therefore assumed to be from the Sinú River basin. Indeed, Oligocene accretionary prism sediments crop out over wide areas of the Sinú River basin and are referred to as Floresanto Formation [38].

The carbonate consists of fine peloidal and detrital micrite, and shows evidence of bioturbation. Pyrite-rimmed dissolution features are common, and there are occasional small voids that lack rim cements (Fig 3C). The carbon isotope values fall within a narrow range of –24.5 to –22.9‰ (Fig 4), indicative of hydrocarbon seepage [36]. The corresponding oxygen isotope values range from –6.3 to –5.7‰ and thus indicate considerable diagenetic alteration and as above, the two Sr isotope measurements (samples Sr-8 and Sr-9) fall below the Sr isotope curve for the Cenozoic (Table 1).

#### Fauna

**Fig 6:**
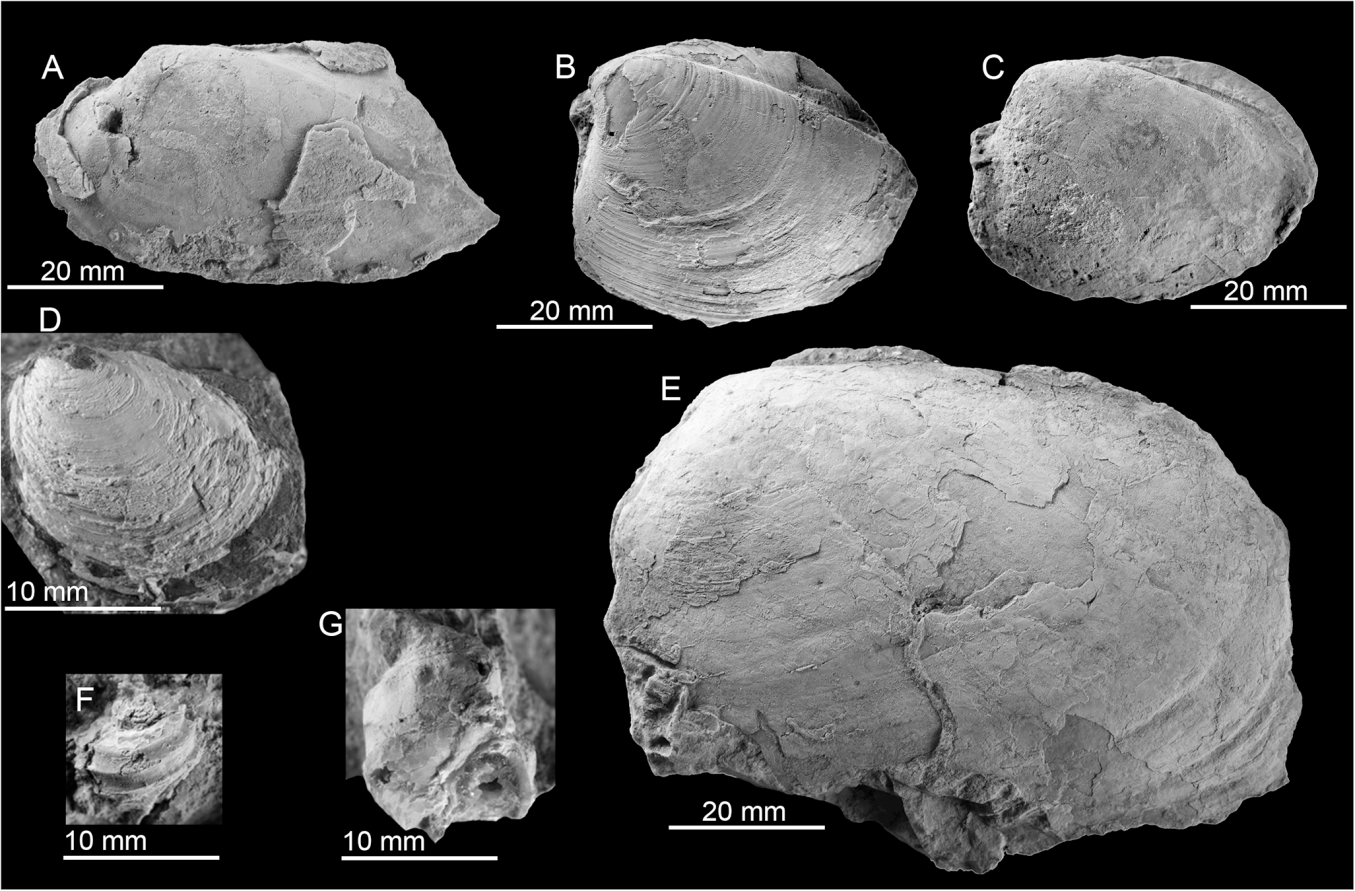
The Oligocene Sta. Clara seep fauna from Colombia. A: The vesicomyid *Pleurophopsis* sp. (PRI 68653). B: The thyasirid *Conchocele adoccasa* (Van Winkle, 1919) (PRI 68654). C: The vesicomyid *Pliocardia* sp. (PRI 68655). D: Oval bivalve resembling *Nucinella*? (PRI 68656). E: The lucinid *Elongatolucina* sp. (PRI x13). F: The seguenzoid *Cataegis godineauensis* (Van Winkle, 1919) (PRI 68657). G: Neogastropod (PRI 68658).

Fossils are preserved either with recrystallized shell or as internal molds with chalky surface. The fauna consists of the vesicomyid bivalves *Pliocardia* sp. (common) and *Pleurophopsis* sp., the lucinid *Elongatolucina* sp., an unidentified, small oval bivalve that externally resembles *Nucinella* Wood, 1851, and the thyasirid *Conchocele adoccasa* (Van Winkle, 1919). This latter species was originally regarded as belonging to *Thyasira* Lamarck, 1818 [21] but its large size, rectangular outline and two posterior ridges and sulcus clearly place this species in *Conchocele* Gabb, 1866 [39, 40]. The two gastropods from this site are a neogastropod and the seguenzoid *Cataegis godineauensis* (Van Winkle, 1919); the latter species was originally regarded as belonging to *Solariella* Wood, 1842, but with its strong spiral sculpture and fine commarginal ribblets it clearly shows affinities to extant species of *Cataegis* [41]. A summary of the fauna is provided in Table 3 and it is illustrated in Fig 6.

**Table 3:** List of taxa from Sta. Clara, Colombia.

Species	Max. size (mm)	N of specimens
Oval bivalve (*Nucinella*?)	22.5	3
*Conchocele adoccasa* (Van Winkle 1919)	72	3
*Elongatolucina* sp.	86	2
*Pleurophopsis* sp.	59.5	2
*Pliocardia* sp.	44	12
*Cataegis godineauensis* (Van Winkle 1919)	7	1
Neogastropod	32	1

## Cuba

Cenozoic seep deposits occur in northwestern Cuba in the vicinity of the capital Havana. A presumably Oligocene site with an unusual fauna consisting of the large, globular gastropod *Elmira cornuatietis* Cooke, 1919, an elongate veneriform bivalve of uncertain affinities described as *Unio*? *bitumen* Cooke, 1919, the lucinid *Cubatea asphaltica* (Cooke, 1919) and possible abyssochrysid gastropods, was reported from the Elmira asphalt mine at Bejucal [15, 34]. Here we report and discuss Sr-isotope ages for the Elmira asphalt mine locality, and describe a new seep fauna of early Miocene age from near the village of Jamaica in the Havana province.

### Elmira asphalt mine site

#### Stratigraphic age

Three Sr isotope measurements were made (Table 1), two from a small calcareous concretion (samples Sr-13 and Sr-14), one from the shell of the bivalve *Unio*? *bitumen* (sample Sr-15). One date from the concretion indicates an upper middle Eocene age (sample Sr-13, Bartonian, 39 Ma), while the other and the bivalve shell indicate an Early Eocene age (samples Sr-14 and Sr-15, late Ypresian, 48.4 to 49.25 Ma). According to the map of French & Schenk [42], both Eocene and Oligocene sediments crop out in the area of Bejucal, and in his original report on the locality, Cooke wrote [22] that “The fossils are doubtfully referred to the Oligocene”, without providing any basis for this assignment. The fossil assemblage at this site is rather unusual compared to those described here and elsewhere for the Caribbean Cenozoic and it may therefore indeed be older than Oligocene. Even the early Eocene age is not contradicted by the fauna: the *Unio*? *bitumen* is as-yet difficult to place taxonomically, the only other occurrence of the gastropod *Elmira* Cooke, 1919 is at a late Cretaceous seep deposit in Japan (Takami Nobuhara and SK, unpublished observation), and gastropods resembling the abyssochrysoid from the Elmira asphalt mine are mostly found in Cretaceous and Eocene seep deposits [43–45].

### Cantera Portugalete

#### Location and stratigraphic age

This material was collected by Dorothy K. Palmer during her pioneering work on the stratigraphy and geology of Cuba. All specimens have the number 802 written onto them, which most likely is Palmer’s locality number. The description of this locality is “Habana Province, Cantera Portugalete, 1 km. N of Jamaica. Ls chips. 1/9/32” [46]. The village of Jamaica is just NW of San José de las Lajas, about 30 km SE of Havana, at 22°58’44” N, 82°10’11” W. The sediments in this area belong to the early Miocene Husillo Formation and represent a “carbonate and carbonate-terrigenous sequence” that was deposited in a deep marine channel between present-day western and central Cuba [47, 48]. The Husillo Formation contains indurated, dark gray, massive rocks [47, 49] like those associated with the fossils concerned here. Two samples for Sr isotope stratigraphy were taken from the carbonate that forms the internal molds of two bivalves (samples Sr-1 and Sr-17), and both indicate a basal Burdigalian age (19.4 to 19.5 Ma), consistent with the early Miocene age of the Husillo Formation (Table 1).

#### Fauna

**Fig 7:**
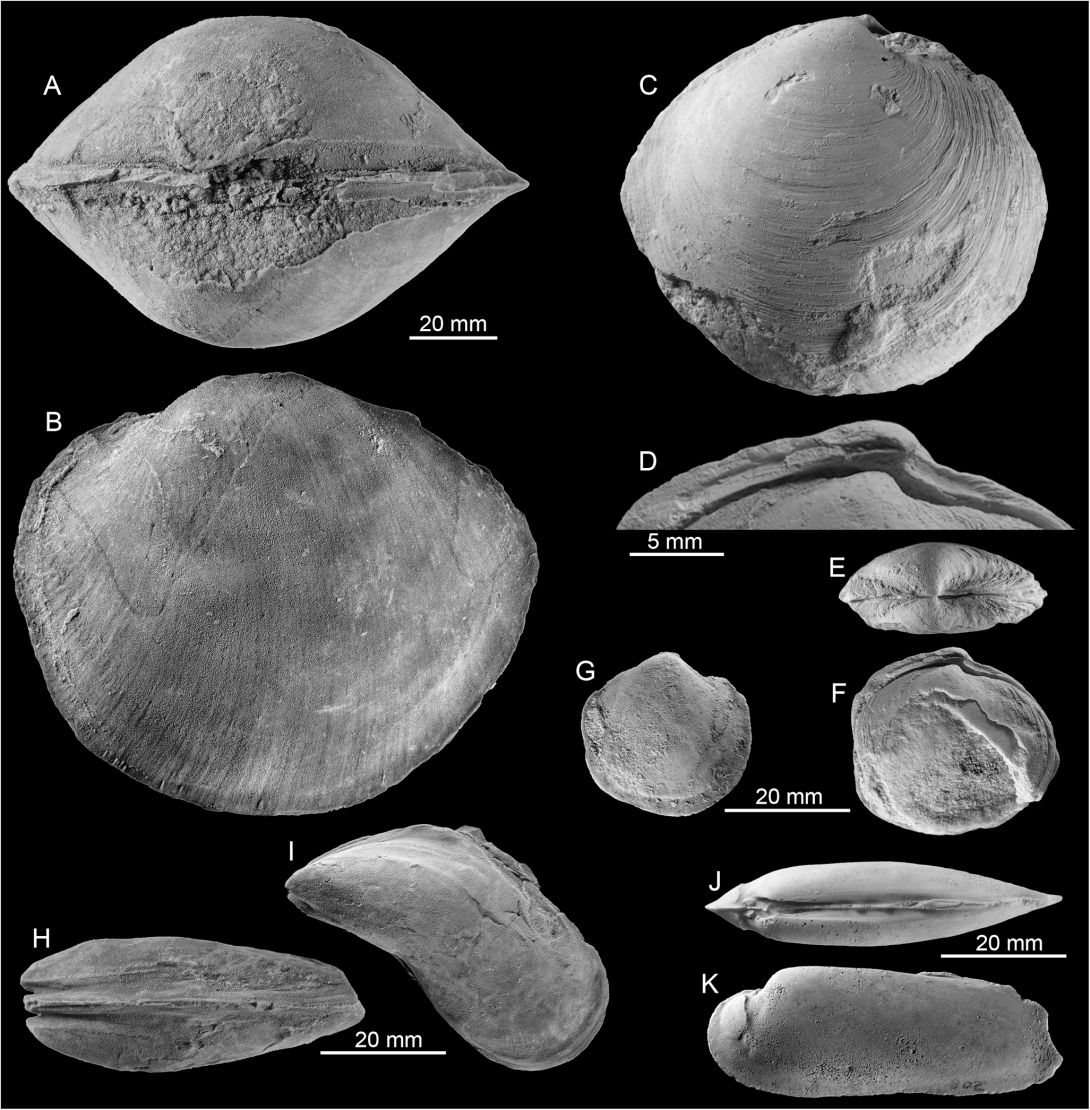
The Early Miocene Cantera Portugalete seep fauna from Cuba. A-C: The lucinid *Meganodontia* sp. (A and B; PRI 68659. C; PRI 68660). D-G: The lucinid bivalve *Myrteopsis*? sp.; three views of a specimen with preserved shell and hinge (D-F; PRI 68661) and internal mold showing adductor muscle scars (G; PRI 68662). H, I: Mytilid (PRI 68663). J, K: *Pleurophopsis* cf. *lithophagoides* Olsson 1931 (PRI 68664).

Few fossils preserve recrystallized shell material, most are internal molds. The most conspicuous species is the large (up to 120 mm long) lucinid bivalve *Meganodontia* sp. The other species are an up to 90 mm long vesicomyid bivalve resembling *Pleurophopsis lithophagoides* Olsson 1931, a small (44.5 mm) lucinid resembling the extant *Myrteopsis*? *lens* Verrill and Smith, 1880 from the western Atlantic Ocean, and a mytilid that reaches 56 mm in length. The mytilid species is unusual among seep-inhabiting mussels because it does not resemble any extant or fossil bathymodiolin, but instead has a terminal umbo and the curved outline of species belonging to *Brachidontes* or *Mytilus*. A species of *Brachidontes* is here reported from a seep deposit at La Piedra in Venezuela (see below), which is also associated with a large *Meganodontia*. The Cuban species may thus also belong to *Brachidontes*. The fauna is summarized in Table 4 and illustrated on Fig 7.

**Table 4:** List of taxa from Cantera Portugalete, Cuba.

Species	Max. size (mm)	N of specimens
*Meganodontia* sp.	120	3
*Myrteopsis*? sp.	44.5	8
*Pleurophopsis aff*. *lithophagoides* Olsson, 1931	90	7
Mytilid	56	5

#### Paleoecology

The carbon isotope signature of the carbonate attached to the fossils or composing the internal molds ranges from –10 to –4.4‰ (Fig 4). These are less negative values compared to the other seep deposits reported herein and are not necessarily indicative of a seep limestone. However, during the precipitation of seep carbonate the typically very negative original isotope signature of the seeping methane is ‘diluted’ by mixing with other carbon sources (i.e., marine bicarbonate) [36]. Considering that (i) the Husillo Formation is largely a carbonate sequence the addition of marine carbonate was likely to be very high, and (ii) the fauna consists largely of chemosymbiotic bivalves, the Cantera Portugalete fauna is here interpreted to have lived at a methane seep, possibly with low rates of seepage and diffuse fluid flow.

## Dominican Republic

### Location and stratigraphic age

The material from NMB loc. 16813 is from the Gurabo Formation, found in the Cañada de Zamba of the Río Cana, in the Cibao Valley in the northern Dominican Republic. The Gurabo Formation in the Río Cana section has been assigned an age that ranges from 5.7 to 4.3 Ma, mostly early Pliocene [50], and the section with Basel loc. 16813 is in the lower part of the Gurabo Formation, very close to the Miocene-Pliocene boundary (Donald McNeill, pers. comm. 2015).

### Fauna and paleoecology

**Fig 8:**
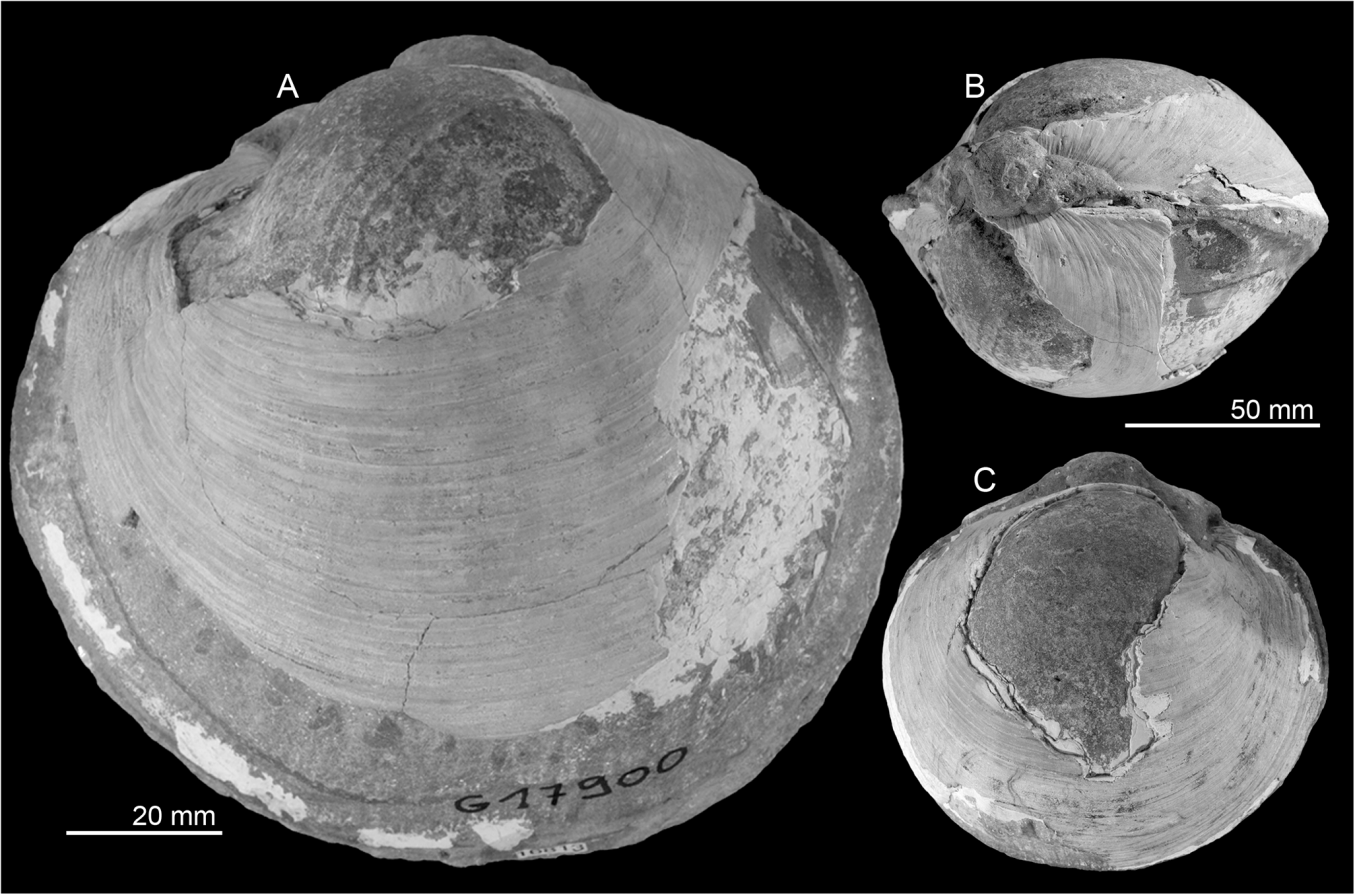
The early Pliocene seep-cemented lucinid bivalve *Anodontia* (*Pegophysema*) sp. from Cañada de Zamba, Dominican Republic. A: view on left valve showing the very elongate anterior adductor muscle scar and the impression of the pallial blood vessel (NMB 17900). B, C: same specimen as in Fig A, in dorsal view (B) and view on right valve (C).

The fauna consists of numerous large (up to 115 mm long) specimens of *Anodontia* (*Pegophysema*) sp., possibly a precursor to the very similar Recent Caribbean *A*. (*P*.) *schrammi* (Crosse, 1876) (Fig 8). One of the specimens has some hard micritic matrix adhering to it, which shows a carbon isotope signature of –28 to –20‰ (Fig 4). Especially the lower value indicates the oxidation of hydrocarbons including methane during the formation of this carbonate.

## Trinidad

**Fig 9:**
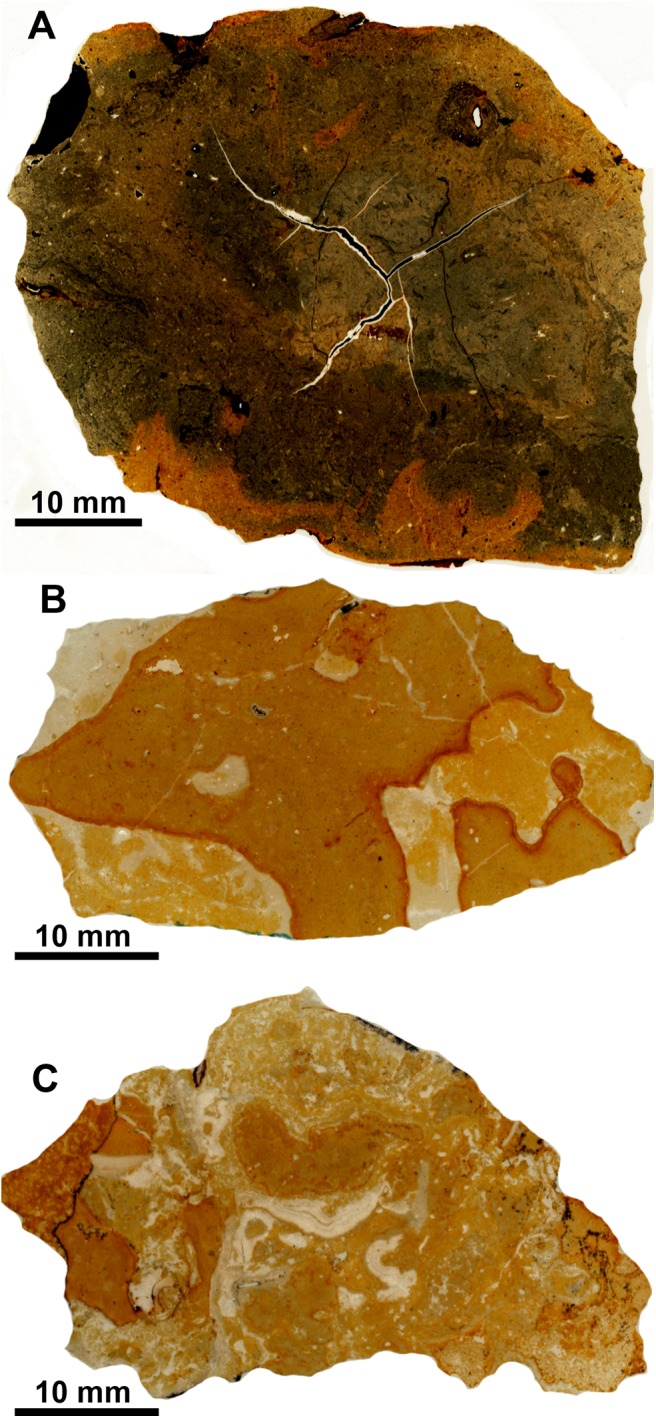
Petrography of the seep carbonates from Trinidad. Scanned thin sections. A: Godineau River. B: Freeman’s Bay. C: Jordan Hill.

The Cenozoic seep faunas of Trinidad reported so far were found along the shore of Freeman’s Bay to the West of San Fernando, in the vicinity of the mouth of Godineau River. Here they were collected as float material on the beach, as mentioned in various reports [14, 20, 21, 51] and indeed, many of the samples investigated here have numerous small extant barnacles attached to them. The fossils were reported to be associated with sediments of the Lengua Formation, which were either considered as middle Miocene [52] or late Miocene [53]. They were reported from two different lithologies (Fig 9) associated with two different preservational styles: (i) from a large, isolated, impure limestone block on the costal mudflat where fossils occur as internal molds, and (ii) from often brecciated, oil-impregnated calcareous mudstone lenses surrounded by greenish, unctuous and slickensided clays in the adjoining coastal section where the fossils occur as black, oil-impregnated specimens often with original shell material [14, 51]. The impure limestone block was given its own lithostratigraphic unit, the “Freeman’s Bay limestone Member”, the oil-impregnated calcareous mudstone lenses were interpreted as having formed at mud diapirs [14]. Despite these differences, paleoecologic interpretations were considered as difficult to reach and the available fossils were reported as a single seep fauna [14].

Based on the material available at the NMB and the PRI, we separate two ecologically distinct types of seep faunas in the Miocene of Trinidad: the dark, bitumen-impregnated fauna is coined ‘Godineau River fauna’ because the boxes containing this fauna at the PRI were labeled Godineau River, whereas the fauna associated with the white limestone is referred to as ‘Freeman’s Bay fauna’. Two further collections with a typical Freeman’s Bay fauna were recovered, Bronte Estate and Jordan Hill, from an area to the East of San Fernando, towards Princess Town. Our Sr-isotope work indicates a late Miocene age for the Godineau River and Jordan Hill faunas (Table 1), which supports the stratigraphic scheme of Erlich et al. [53].

### Godineau River fauna

#### Stratigraphic age

Two Sr isotope measurements were made: one sample was from the calcareous mudstone and indicates a middle Messinian age (sample Sr-19, 6.35 Ma), the other is from the aragonitic shell of the possible vesicomyid bivalve, which gave a basal Tortonian age (sample Sr-18, 11.1 Ma). The corresponding oxygen isotope value of the latter sample (Table 1) indicates slight diagenetic alteration of this material by meteoric water, thus the Messinian age derived from the apparently unaltered calcareous mudstone is here considered the more reliable. However, both dates suggest a late Miocene age for the Godineau River fauna.

#### Fauna and paleoecology

**Fig 10:**
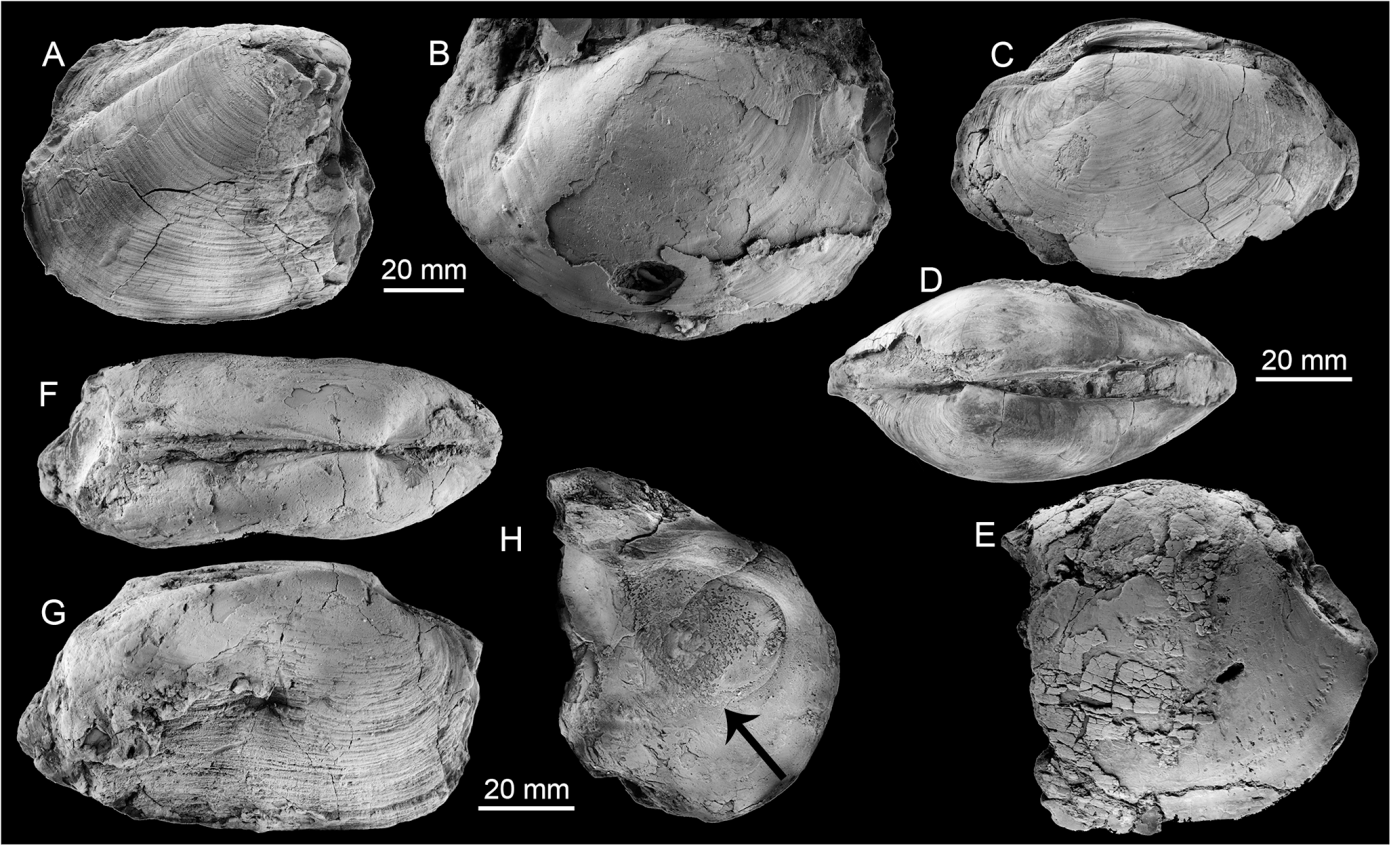
The late Miocene Godineau River seep fauna in Trinidad. A: The thyasirid *Conchocele adoccasa* (Van Winkle, 1919) (PRI 68665). B: The lucinid *Nipponothracia* sp. (PRI 68666). C-E: The lucinid *Elliptiolucina* sp.; specimen with preserved shell and hinge (C, D; PRI 68667), internal mold showing anterior adductor muscle scar (E; PRI 68668). F-H: A possible vesicomyid; external view (F, G; PRI 68669) and interior of fragment showing anterior adductor muscle scar (H; PRI 68670).

**Fig 11:**
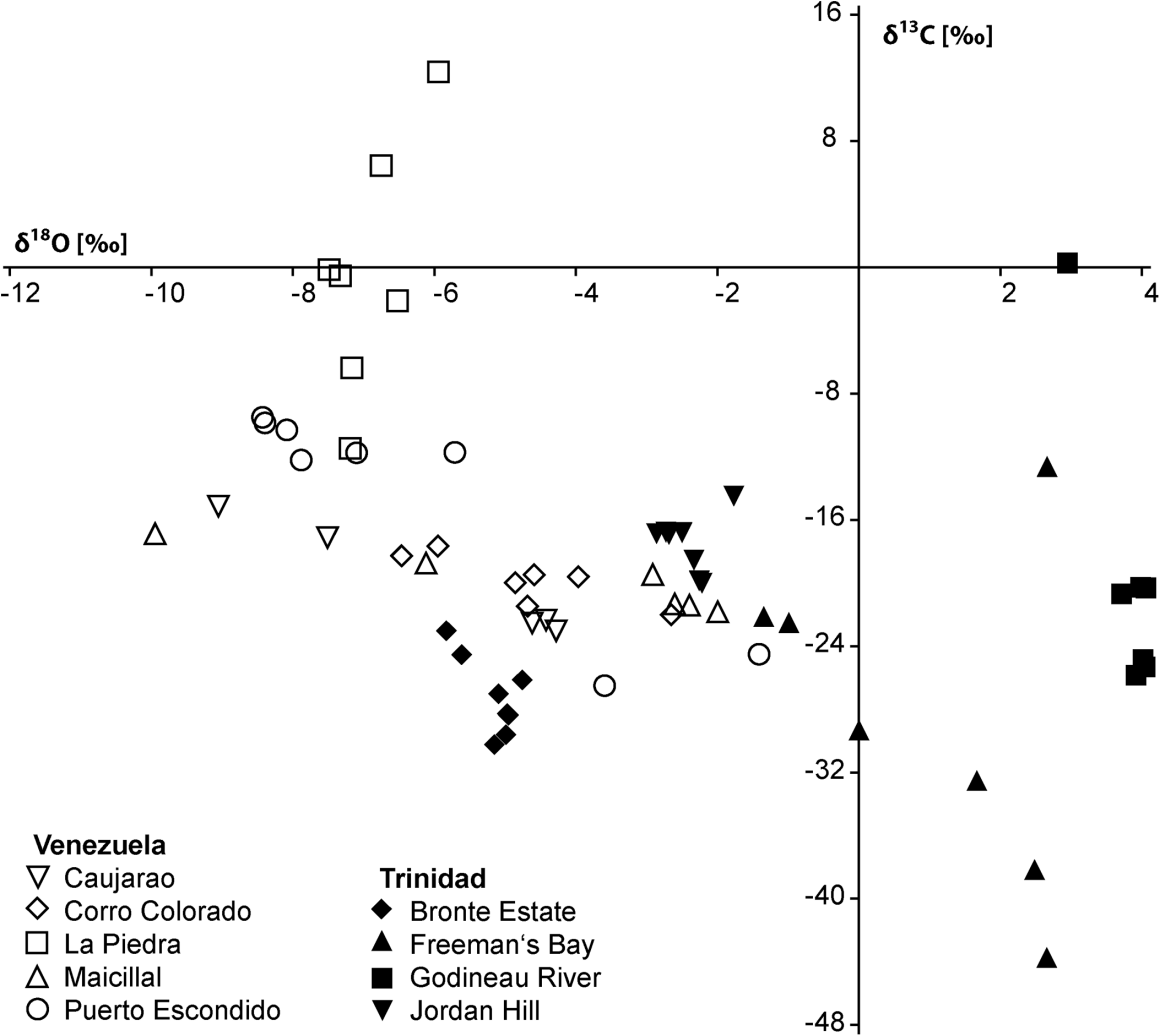
Cross-plot of stable carbon and oxygen isotope data. Filled symbols: Trinidad, open symbols: Venezuela.

The Godineau River fauna consists exclusively of very large, infaunal or semi-infaunal chemosymbiotic bivalves (Table 5, Fig 10): the thyasirid *Conchocele adoccasa* reaching 87 mm length, the lucinids *Nipponothracia* sp. reaching 167 mm in length and *Elliptiolucina* sp. reaching 85 mm length, and an unidentified species that may (or may not) belong to the Vesicomyidae, reaching 140 mm length. In thin section, the oil-impregnated calcareous mudstone is composed of monotonous micrite with rare planktonic foraminiferans (Fig 9A). Its carbon isotope signature ranges from –25.8 to –20.2‰, with corresponding oxygen isotope values ranging from 3.7 to 4‰ (Fig 11), indicating that the limestone formed due to methane oxidation with a considerable amount of marine bicarbonate mixed in [36].

**Table 5:** List of taxa from Godineau River, Trinidad.

Species	Max. size (mm)	N of specimens
*Conchocele adoccasa* (Van Winkle 1919)	87	8
*Nipponothracia* sp.	167	8
*Elliptiolucina* sp.	85	2
Vesicomyid? sp.	140	8

### Freeman’s Bay

#### Stratigraphic age

Two Sr isotope measurements were made on two differently colored micrites (samples Sr-20 and Sr-21, Table 1). The accompanying oxygen isotope values were slightly negative, suggesting diagenetic alteration by meteoric waters, and the ages derived from the Sr isotope ratios were younger than reasonable considering the biostratigraphic framework of this locality.

#### Fauna and paleoecology

**Fig 12:**
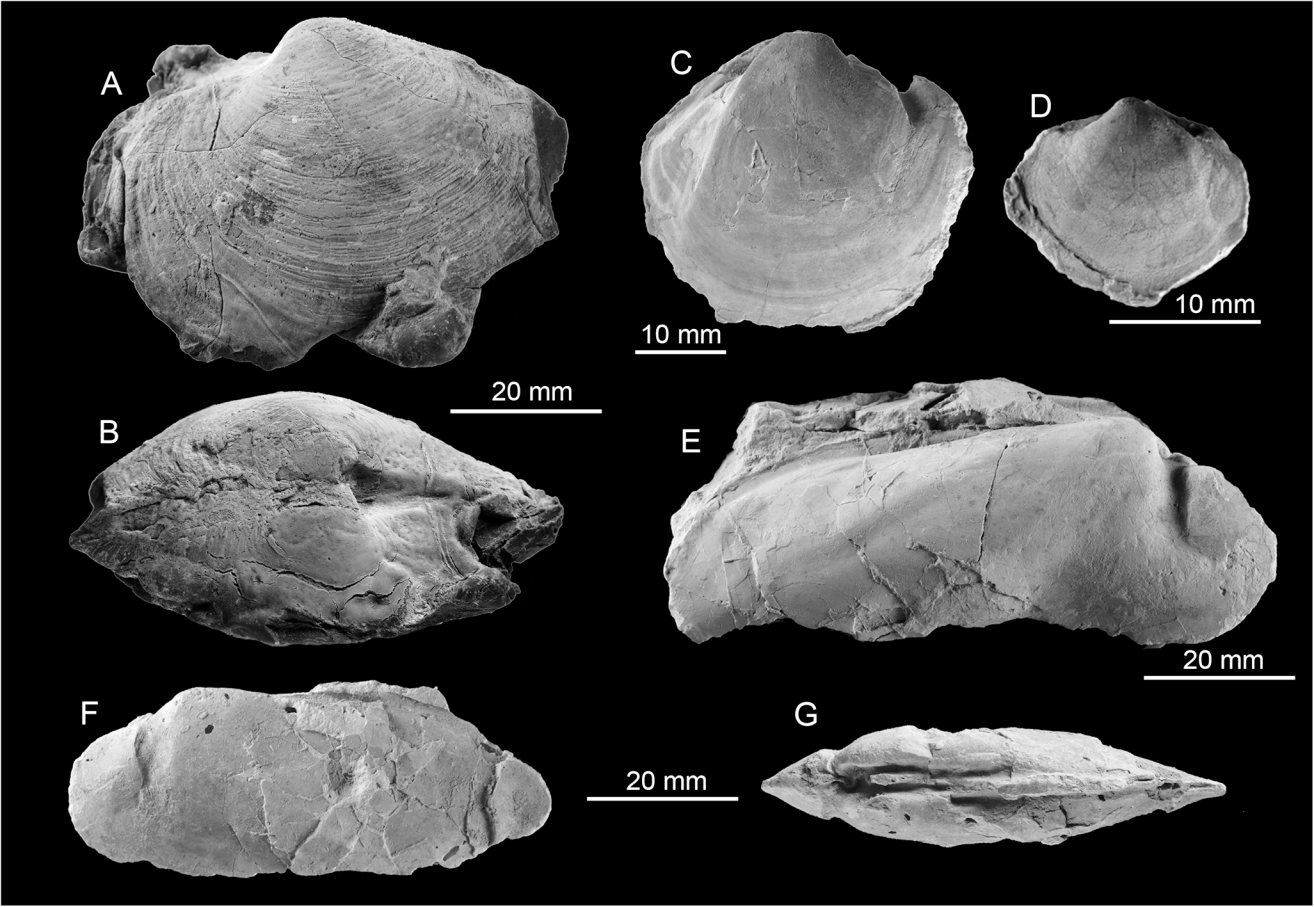
Examples of the late Miocene Freeman’s Bay seep fauna from Trinidad. A, B: The lucinid *Cubatea* sp. (PRI 68671). C, D: Lucinid bivalve (PRI 68672). E: The vesicomyid *Pleurophopsis unioides* var. *fernandensis* Van Winkle, 1919 (PRI 68673). F, G: *Pleurophopsis unioides* Van Winkle, 1919 (PRI 68674).

The fauna consists largely of the vesicomyid bivalve *Pleurophopsis unioides* Van Winkle, 1919 and *P*. *u*. var. *fernandensis* Van Winkle, 1919 that reach just over 80 mm length. The validity of the genus *Pleurophopsis* had been questioned because of its poorly preserved type material [54], but was considered valid by other authors [55, 56]. The material investigated during the present study indicates that it is indeed a valid and distinctive genus; further details will be published in a forthcoming paper. Minor elements of the Freeman’s Bay fauna include the lucinid bivalves *Cubatea* sp. and an unidentified lucinid, which are here reported for the first time from this fauna, *Conchocele adoccasa*, which is the only species shared between the Freeman’s Bay and Godineau River faunas, a nuculanid bivalve, and four gastropods: *Cataegis godineauensis* (Van Winkle 1919), *Cantrainea* sp., *Provanna* sp., and a limpet (Table 6, Fig 12). In addition, a bathymodiolin mussel has been reported [14, 21].

**Table 6:** List of taxa from Freeman’s Bay, Trinidad.

Species	Max. size (mm)	N of specimens
Nuculanid	7	2
*Conchocele adoccasa* (Van Winkle 1919)	21	1 external mold
*Pleurophopsis unioides* Van Winkle 1919	83	ca. 40
*Pleurophopsis u*. var. *fernandensis* Van Winkle 1919	81	5
*Cubatea* sp.	57.5	4
*Cantrainea* sp.	8	2
*Cataegis godineauensis* (Van Winkle 1919)	9	2
*Provanna* sp.	8.5	10
Limpet gastropod	6	1

The Freeman’s Bay fauna was characterized by Van Winkle as “very peculiar and unlike any known” [21] and later interpreted as a fossil seep fauna [14, 57]. Evidence for this interpretation has never been provided, and it was presumably based on the abundance of chemosymbiotic bivalves. The carbon isotope signature of the limestone ranges from –20 to –14.5‰, with corresponding oxygen isotope values ranging from –2.8 to –1.7‰ (Fig 11), consistent with the interpretation that methane oxidation was involved in the formation of the carbonate. Petrographically the Freeman’s Bay limestone shows abundant traces of bioturbation and bioclasts (Fig 9B), unlike the Godineau River material.

### Bronte Estate

#### Location and stratigraphic age

The locality description for NMB locality 10228 reads “Lengua Formation, Freeman’s Bay limestone Member. Naparima area, Bronte Estate, Manager House. Quarry near trigonometric station W of Naparima.” Today there is a ‘Bronte village, Naparima’ just south of the Jordan Hill Presbyterian school, exactly where the Lengua Formation crops out according to Kugler’s map [51]. It is therefore assumed that the fossils reported here were collected in this area. Due to the (assumed) close proximity to the Jordan Hill site, this locality is considered to be of late Miocene age.

#### Fauna and paleoecology

**Fig 13:**
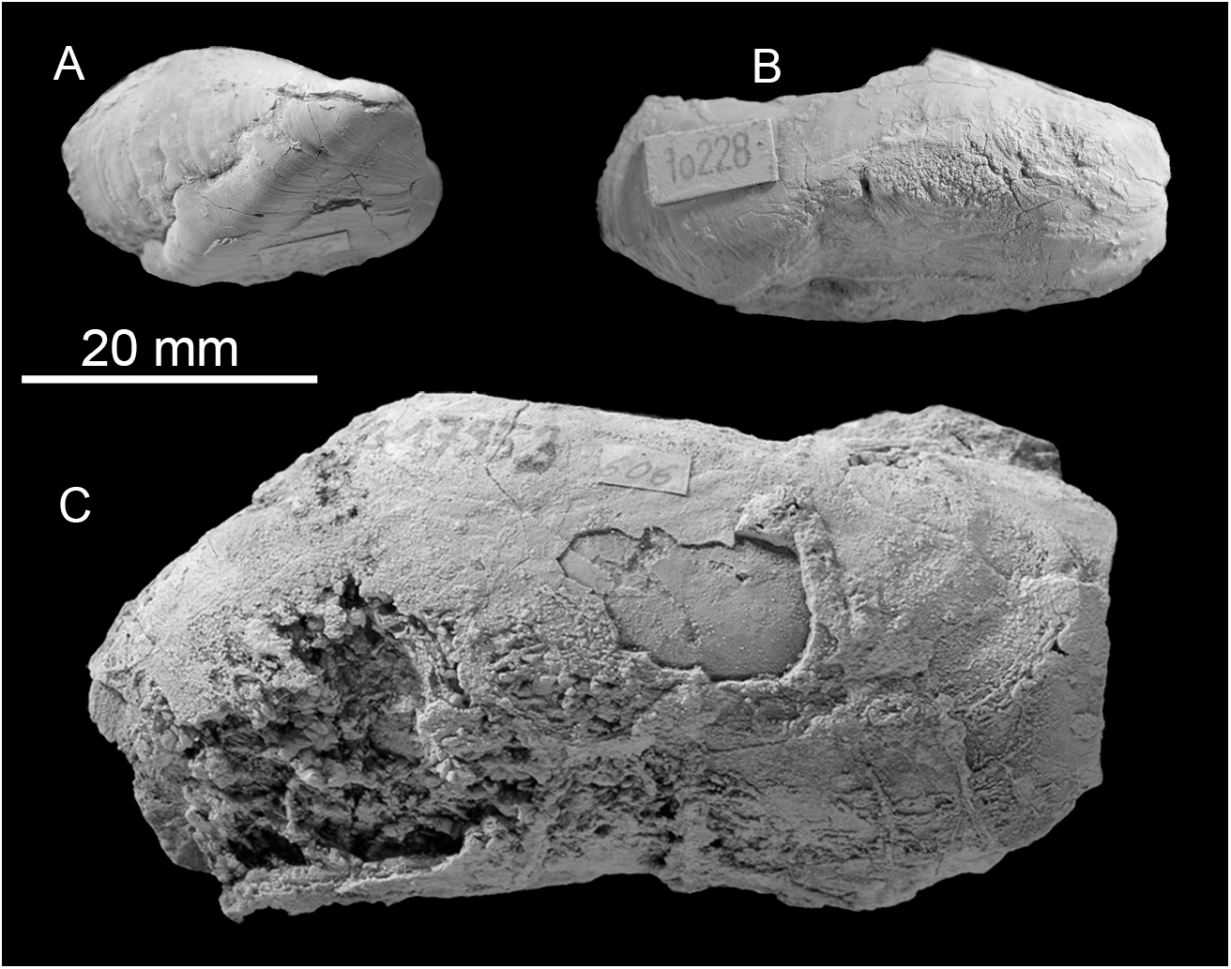
The late Miocene Bronte Estate seep fauna from Trinidad. A: The mytilid *Bathymodiolus* ‘short’ (NMB G17955). B. The mytilid *Bathymodiolus* ‘elongate’ NMB G17956). C: The vesicomyid *Pleurophopsis unioides* var. *fernandensis* Van Winkle 1919 (NMB G17953).

The fauna consists of *Pleurophopsis unioides* var. *fernandensis* reaching 110 mm in length, and probably two *Bathymodiolus* species, the elongate one reaching 40 mm in length, the short one reaching 29.3 mm in length (Table 7, Fig 13). The carbonate adhering to the fossils is whitish-ochre colored micrite; material for thin sectioning was not available. Its carbon isotope signature ranges from –30.2 to –23‰, clearly indicating than methane oxidation was involved in the formation of the carbonate. The corresponding δ^18^O values range from –5.8 to –4.8‰ (Fig 11).

**Table 7:** List of taxa from Bronte Estate, Trinidad.

Species	Max. size (mm)	N of specimens
*Pleurophopsis u*. var. *fernandensis*	110	5
*Bathymodiolus* sp. ‘short’	29.3	6
*Bathymodiolus* sp. ‘elongate’	40	2

### Jordan Hill

#### Location and stratigraphic age

The labels of this small collection read “Jordan Hill House” and “Trinidad GDH”, suggesting that it was collected by Gilbert Dennison Harris. Today there is a Jordan Hill Presbyterian primary school on the Manahambre road, about halfway between San Fernando and Princes Town, at 10°15'22"N, 61°24'29"W, and it is assumed that the material was collected around here. Kugler’s map [51] indicates the upper Cipero Formation in this area, which ranges into the late Miocene [53]. However, the Lengua Formation crops out just a kilometer or so to the SW and SE [51], and could thus also be a source of the material reported here. Two samples for Sr isotope stratigraphy indicate a late Tortonian age (samples Sr-22 and Sr-23, 7.7 and 8.8 Ma).

#### Fauna and paleoecology

**Fig 14:**
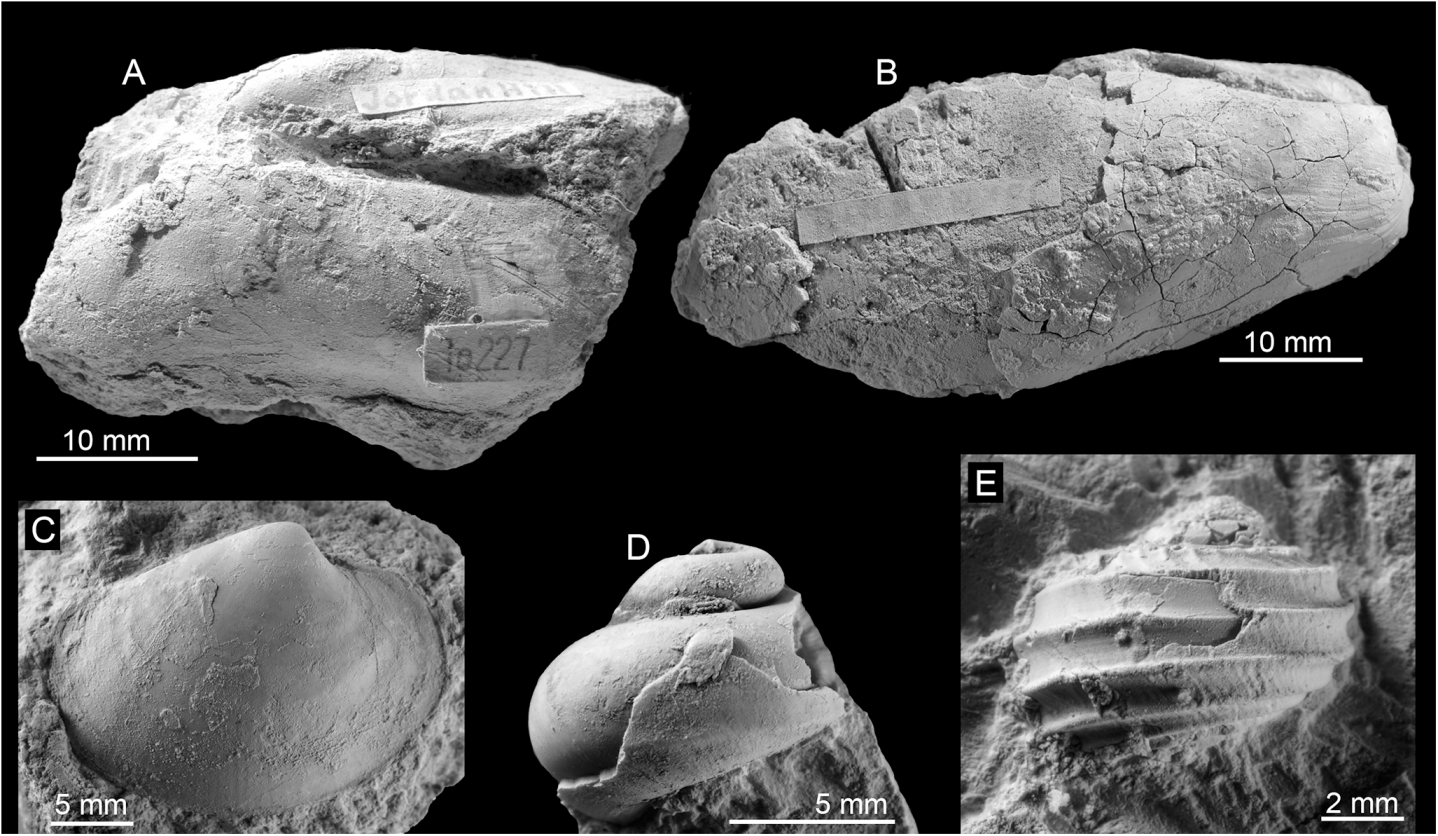
The late Miocene Jordan Hill seep fauna from Trinidad. A: The vesicomyid *Pleurophopsis unioides* var. *fernandensis* Van Winkle, 1919 (NMB G17945). B: The mytilid *Bathymodiolus* aff. *palmarensis* Kiel, Campbell & Gaillard, 2010 (NMB G17946). C: The possible vesicomyid bivalve (PRI 68675). D: The colloniid *Cantrainea* sp. (PRI 68676). E: The seguenzoid *Cataegis godineauensis* Van Winkle, 1919 (PRI 68677).

The fauna (Table 8, Fig 14) is very similar to the Freeman’s Bay fauna except for a small, possible vesicomyid bivalve having the general outline of the genus *Isorropodon* Sturany, 1896 (Fig 14C). Today this genus has a wide geographic distribution but has so far not been reported from the western Atlantic Ocean [58]; the genus was also present in an early Miocene seep deposit in the northeastern Pacific Ocean [59]. The lithology of the Jordan Hill seep deposit differs from both the Freeman’s Bay and Bronte Estate seep deposits by containing more diverse carbonate phases and especially by the presence of various vugs lined with banded rim cements; such cements have not been seen in the Freeman’s Bay and Bronte Estate deposits (Fig 9C). Furthermore, the Jordan Hill seep carbonate shows a wider range in its carbon isotope signature, ranging from –43.7 to –12.3‰ (Fig 11), consistent with its more diverse carbonate phases. The very negative carbon isotope signature clearly indicates that biogenic methane (rather than thermogenic methane) was involved in the formation of the carbonate [36].

**Table 8:** List of taxa from Jordan Hill, Trinidad.

Species	Max. size (mm)	N of specimens
*Pleurophopsis unioides* var. *fernandensis* Van Winkle 1919	110	1
*Bathymodiolus* cf. *palmarensis* Kiel et al. 2010	30	7
Vesicomyid bivalve	26	1
*Cataegis godineauensis* (Van Winkle 1919)	9	1
*Cantrainea* sp.	12.5	1

## Venezuela

Cenozoic seep faunas in Venezuela are found in the Falcon Basin at or near the present-day coast of the Caribbean Sea, and here the moderate to deep-water deposits within the Agua Salada Group. A single Cenozoic seep deposit has so far been reported, from Puerto Escondido in Estado Falcón, consisting of solemyid, bathymodiolin, thyasirid, lucinid and vesicomyid bivalves, and a few gastropods, considered as being of Miocene age [14, 60]. The new material reported here is from the collection of the NMB and includes four new sites and new data on the Puerto Escondido site reported earlier [14]. All new sites are presumed to be from the Agua Salada Group in the Estado Falcón, including three from the Pozón Formation, one from the Guacharaca Formation, and one from the Riecito Limestone. They are considered to range in age from Early Oligocene to middle Miocene. Most locality data are from the NMB locality register, addition information and opinions were provided by Oliver Macsotay (pers. comm. 2014), a specialist of the Cenozoic stratigraphy of Venezuela [61].

### Paleoecology

The Venezuelan fossils in the NMB collection had very little or no carbonate matrix adhering to them, thus thin section preparation was not possible and only carbon and oxygen isotope data are available for environmental reconstructions. These isotope data were very similar among the sites (with the exception of La Piedra, discussed below). Therefore, their implications are summarized here, instead of reiterating them at each site description. The δ^13^C values range from –26.5 to –9.5‰, with the majority of values between –22 and –15‰ (Fig 11). These values indicate carbonate precipitation induced by the oxidation of hydrocarbons, but are not necessarily indicative of methane as sole hydrocarbon [15, 36]. However, the characteristic, very negative δ^13^C signature of methane can be diluted by marine carbonate in carbonate-rich settings [62]. The Venezuelan sites reported here were formed in tropical marl- and limestone, middle to outer shelf settings where a strong input in marine carbonate can be expected. Hence we assume that the sites reported here formed at ancient hydrocarbon seeps where methane formed an important part of the hydrocarbons.

### Buenavista de Maicillal

**Fig 15:**
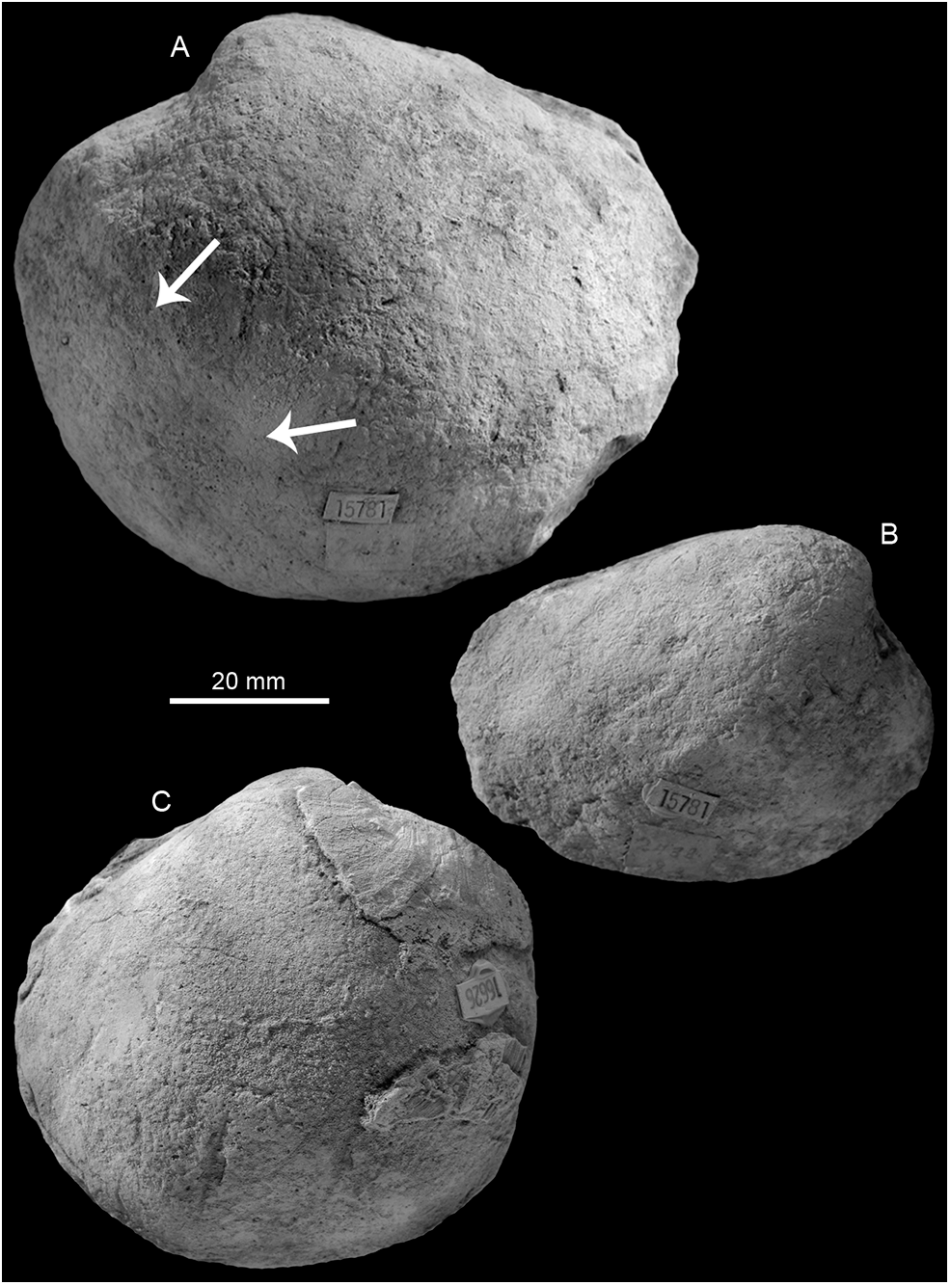
The Oligocene to Miocene Buenavista de Maicillal and Caujarao seep faunas from Venezuela. A: A large lucinid bivalve from Buenavista de Maicillal (NMB G18007). B: A possible vesicomyid bivalve from Buenavista de Maicillal (NMB G18008). C: A large lucinid bivalve from Caujarao (NMB G17996).

This small collection (Basel loc. 15781) obtained by Paul Leuzinger is from Buenavista de Maicillal in the Mirimire area in the Estado Falcón. It belongs to the Pozón Formation of the Agua Salada Group and here to “its Husite member, composed of marly clays interbedded with foraminifera marls, with scattered glauconite. The paleodepth based on ostracods is 100–170 m [63]” (Oliver Macsotay pers. comm. 2014). Its age is given as ‘mid-late Miocene?’ (label), as middle to late Oligocene (NMB locality register) or early Middle Miocene (Oliver Macsotay pers. comm. 2014). Three samples from the micritic matrix were used for Sr isotope stratigraphy (samples Sr-26 to Sr-28) and suggest an Aquitanian age (Table 1). The fauna consists of a large, globular lucinid bivalve apparently with a thin, elongate anterior adductor muscle scar, and a possible vesicomyid bivalve (Table 9, Fig 15).

**Table 9:** List of taxa from Buenavista de Maicillal, Venezuela.

Species	Max. size (mm)	N of specimens
Vesicomyid bivalve	61	1
Large, globular lucinid	86	1

### Caujarao

This collection (Basel loc. 16626) is from Río Mirimire near Caujarao in the Aguide area in Estado Falcón. According the NMB locality register it belongs to the Guayabal marls of the Guacharaca Formation. According to Oliver Macsotay (pers. comm. 2014) “This locality belongs to the La Danta member ([61], p. 336–337), the lowest member of the Guacharaca Fm. This unit is essentially pelitic, with fine-grained turbidites, of 235–250 m in thickness, and should be of Early Oligocene age.” A single measurement of Sr isotope ratios (sample Sr-29) suggests an uppermost Aquitanian (early Miocene) age; however, the oxygen isotope signature of the samples indicates some diagenetic alteration (Table 1). The fauna consists of ten moderately sized (up to 77 mm long) specimens of a lucinid bivalve possibly belonging to *Meganodontia* (Fig 15).

### Corro Colorado

**Fig 16:**
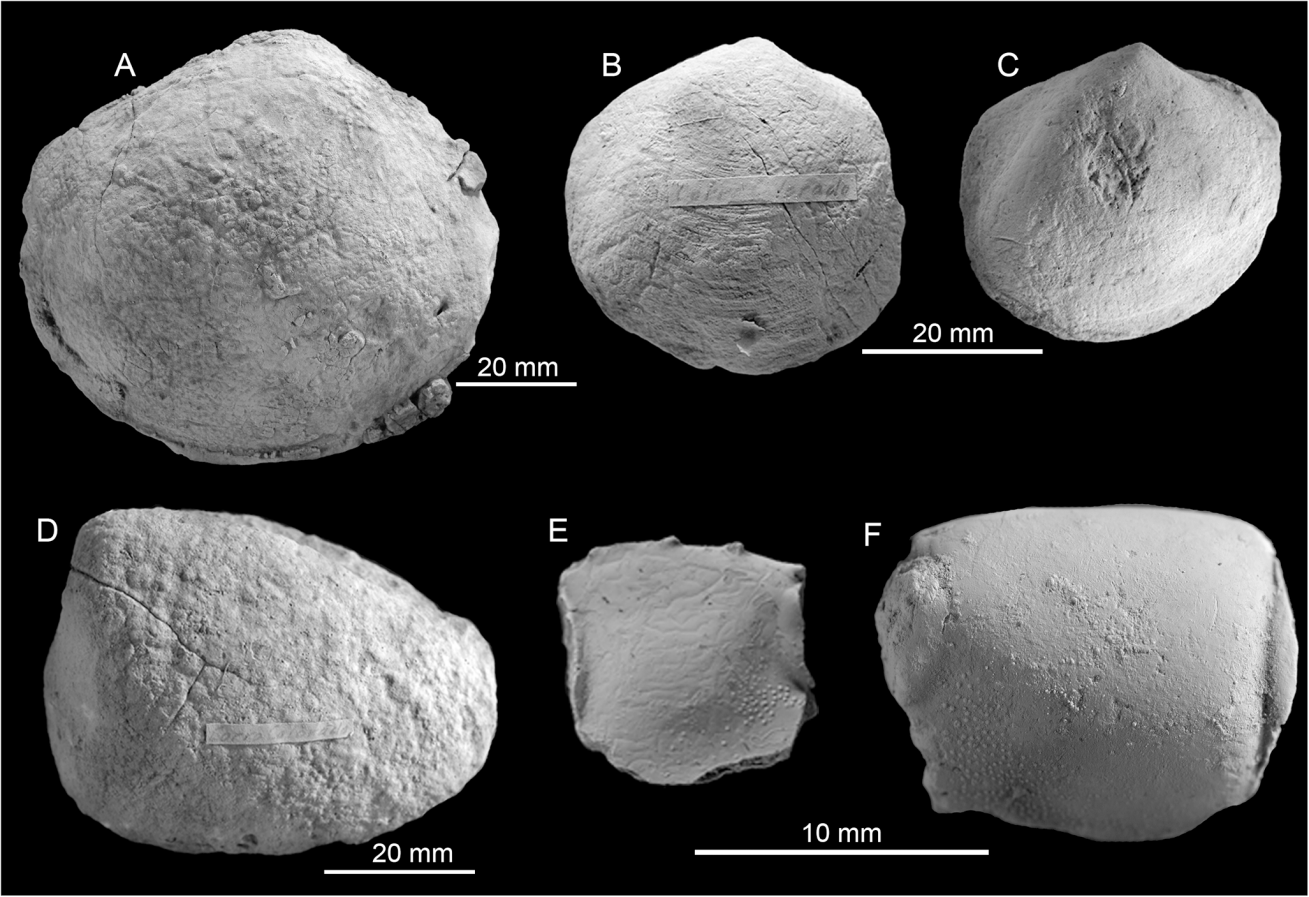
The early to middle Miocene Corro Colorado seep fauna from Venezuela. A: The large lucinid bivalve (NMB G17995). B, C: The small lucinid bivalve (NMB G17992, 17993). D: The possible vesicomyid bivalve (G17965). E, F: Claw fragments of the callianassid ghost shrimp *Glypturus* (NMB G17967, 17968).

The labels associated with this locality read “Corro Colorado, near Maicillal de la Costa, due South on road”and “[unreadable] between Corro Colorado onto road to San Francisco“. According to Oliver Macsotay (pers. comm. 2014) “this locality, by cartography, belongs to the Husite Member of the Pozón Formation. The age is Early Middle Miocene.” Two samples (Sr-24 and Sr-25) were used for Sr isotope stratigraphy and suggests an uppermost Aquitanian to lower Burdigalian (early Miocene) age, and there is only a slight chance of diagenetic alteration as indicated by the oxygen isotope signature of only –2.7‰ (Table 1). The fauna includes a relatively large potential vesicomyid bivalve, a large lucinid bivalve, a smaller lucinid, and abundant callianassid shrimp fragments belonging to the genus *Glypturus* [64] (Table 10, Fig 16).

**Table 10:** List of taxa from Corro Colorado, Venezuela.

Species	Max. size (mm)	N of specimens
*Glypturus* fragments	18	25
Possible vesicomyid bivalve	61	2
Large lucinid bivalve	80	1
Small lucinid bivalve	38	3

### La Piedra

#### Location and stratigraphic age

This small collection (NMB loc. 15780) obtained by Louis Vonderschmidt is from La Piedra in the Isidro area in Estado Falcón. It belongs to the Pozón Formation of the Agua Salada Group and was regarded as either middle to late Oligocene (NMB locality register) or mid-late Miocene (label). Oliver Macsotay (pers. comm. 2014) believes that “the fossils came from Policarpo member, composed of marly limestones, clays, with glauconititc beds at its base. Iron concretions are frequent. […] The paleodepth data given by [65] based on benthic foraminifera should extend from 200 to 600 meters.” The Policarpio “Greensand” Member is of late Aquitanian (early Miocene) age [65]. Two samples (Sr-30 and Sr-31) were used for Sr isotope stratigraphy and broadly suggest a late Miocene age (lower Tortonian to Messinian; Table 1), but with δ^18^O values as low as –6.5‰ the samples appear diagenetically altered and hence the Sr isotope age should be treated cautiously.

#### Fauna and paleoecology

**Fig 17:**
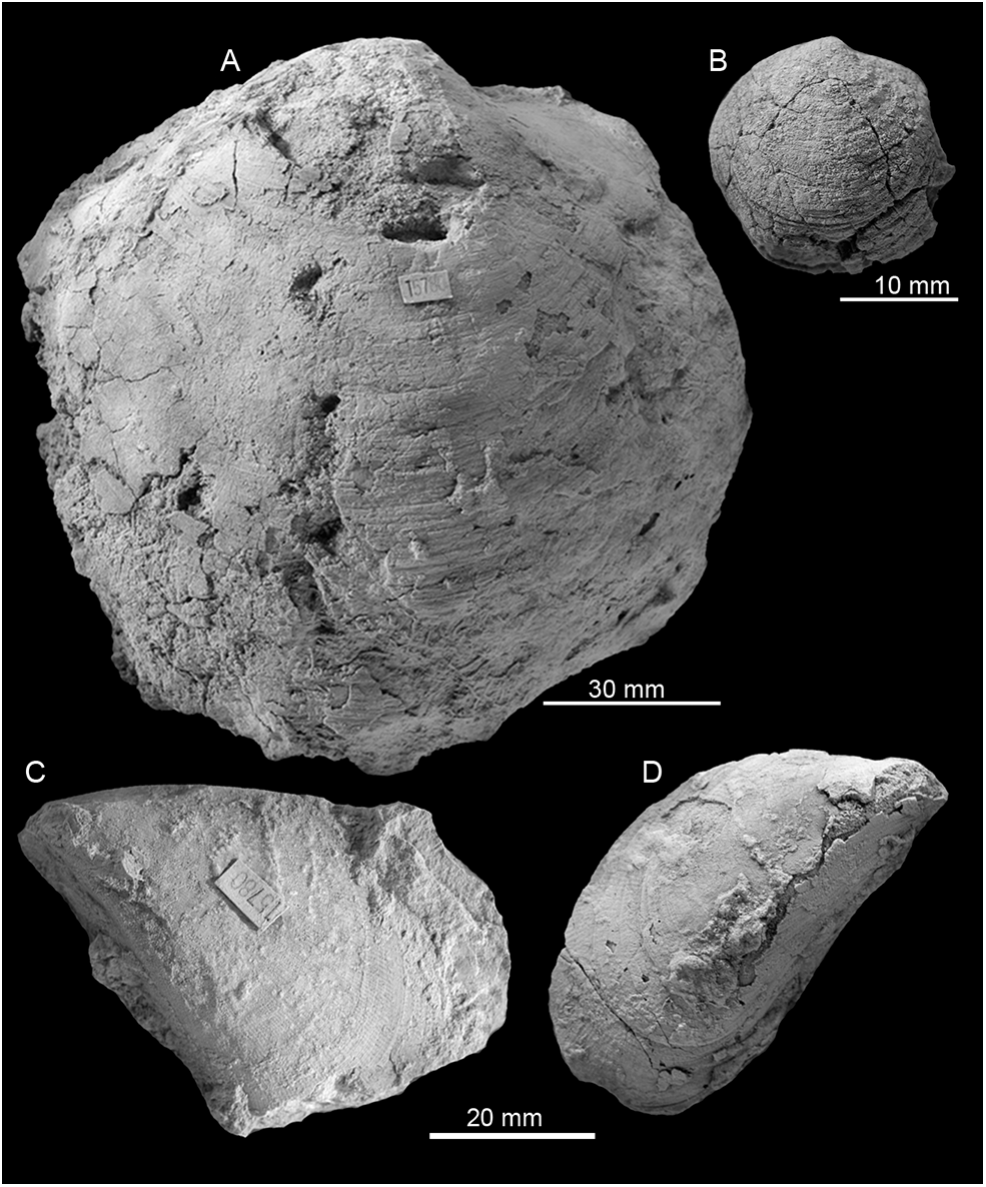
The Miocene La Piedra seep fauna from Venezuela. A: The lucinid *Meganodontia*? sp. (NMB G18003). B: Small lucinid bivalve (NMB G18001). C, D: The mytilid *Brachidontes* sp. (NMB G18005, G18006).

The fauna consists of a large lucinid resembling *Meganodontia*, a small lucinid, and a mytilid belonging to *Brachidontes* (Table 11, Fig 17). The carbon isotope values of the associated carbonate are very heterogeneous, ranging from –11.5 to +12.4‰, with more homogenous corresponding oxygen isotope values between –7.5 to –5.9‰. While the negative carbon isotope values indicate the oxidation of hydrocarbons, the very positive values can only be explained my local methane formation, because the CO_2_ pool utilized during archaeal methanogenesis becomes enriched in ^13^C [36, 66, 67]. Such ^13^C-enriched carbonate phases are interpreted as having formed after the precipitation of the ^13^C-depleted carbonate phases, probably during early burial [36].

**Table 11:** List of taxa from La Piedra, Venezuela.

Species	Max. size (mm)	N of specimens
*Meganodontia*? sp.	125	2
Small lucinid bivalve	21	1
*Brachidontes* sp.	60	3

### Puerto Escondido

The carbonate adhering to a vesicomyid and a *Bathymodiolus* shell from this locality (NMB loc. 13968) has a carbon isotope signature as low as –26.5‰ (Fig 11), supporting the suggestion of [14] that the Puerto Escondido fauna formed at an ancient methane seep deposit. The age of this fauna was given as ‘Miocene’ [14] and it occurs in the Huso Clay Member of the Pozon Formation, which, according to the stratigraphic chart of Blow [65] is late Burdigalian (late Middle Miocene). Two samples (Sr-32 and Sr-33) were used for Sr isotope stratigraphy and both suggest a Messinian age (upper Late Miocene; Table 1).

## Discussion

### Stratigraphy of the Caribbean seep faunas

**Fig 18:**
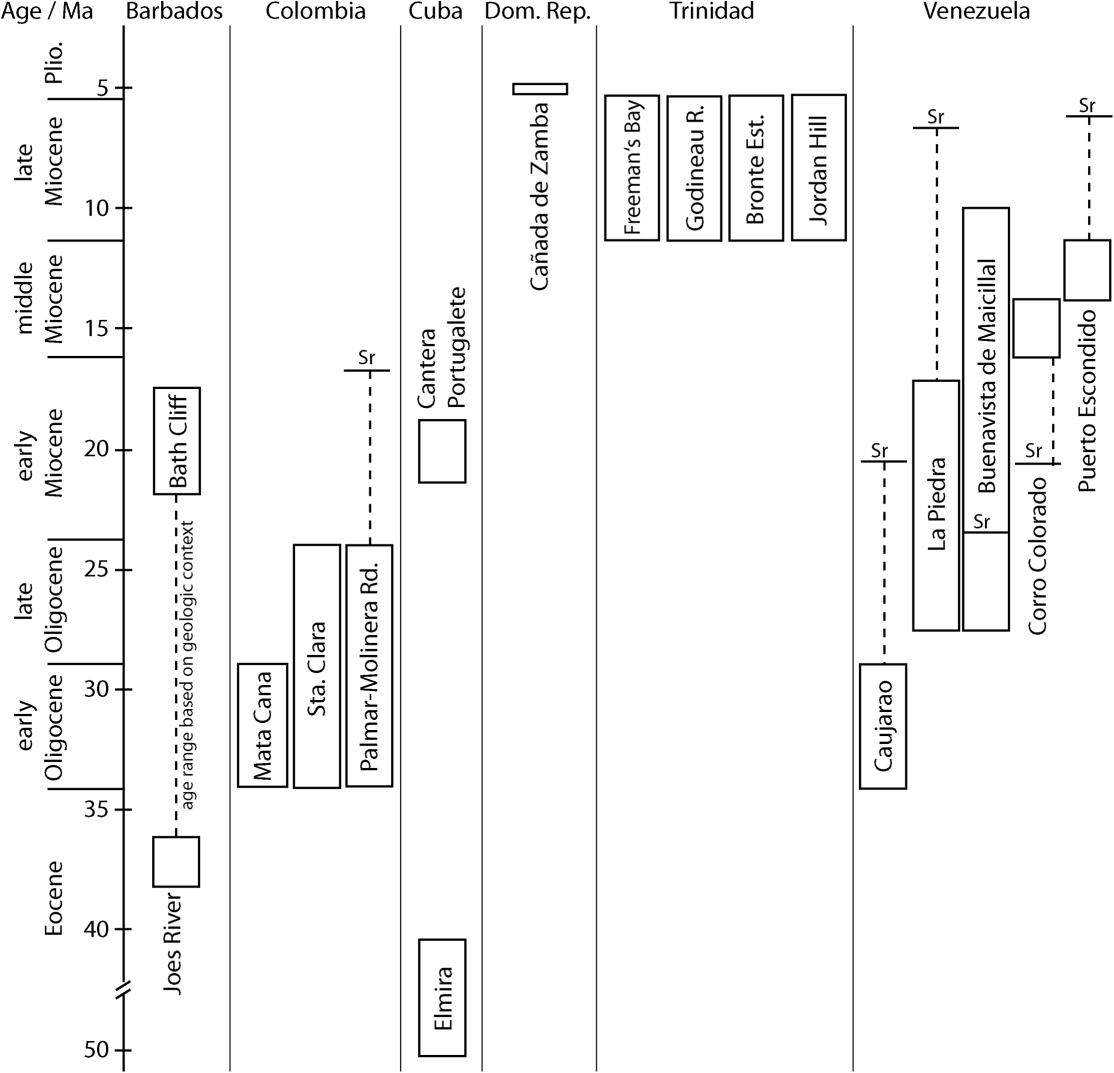
Summary of the geologic ages of the Cenozoic seep faunas from the Caribbean region. Long range indicates uncertainty in dating; note break in time scale below 40 Ma. Sr associated with stippled lines and whiskers indicate minimum or maximum age based on strontium isotope stratigraphy.

The exact geographic location and therefore also the stratigraphic ages of many of the Caribbean seep communities are still not well constrained, and our current understanding is summarized in Fig 18. Although our Sr isotope dating of known and new sites provided new insights into their stratigraphic ages, it also revealed a number of persistent problems and uncertainties.

The Cuban Elmira asphalt mine site, hesitantly considered Oligocene in previous papers [15, 22], may be as old as early Eocene, based on our Sr isotope stratigraphy. This would make it the oldest seep community of the Caribbean region so far. An Eocene age would be consistent with the presence of the relatively large-sized abyssochrysoid gastropods at this site, which occur also the late Eocene ‘Joes River fauna’ of Barbados [24], and are generally a common feature of late Mesozoic to Eocene seep faunas [9, 33, 34, 43, 68].

Fossil seep faunas from Barbados are known from two different rock units: the mudrock-hosted Joes River fauna from the diapiric mélange, and the carbonate-hosted Bath Cliffs fauna from the Suboceanic Fault Zone [14]. These faunas are taxonomically distinct and were recently considered to be of any age between the Eocene and the Miocene, based on the well-established ages of the enclosing units [14]. Our Sr isotope data, although limited, indicate that at least some of these faunas are also stratigraphically distinct: our samples of the Joes River fauna with its large lucinid bivalves and the tall abyssochrysoid gastropods indicate a late Eocene (basal Priabonian) age, and an early Miocene age for the Bath Cliffs fauna with *Pleurophopsis*, *Cataegis*, and associated taxa. However, considering that seep faunas that are ecologically similar to the Joes River and Bath Cliffs faunas, respectively, occur together in late Miocene sediments in Trinidad indicates that these different faunal types may just be the result of different ecological settings [14]. Thus, we cannot exclude the possibility that mudstone- and carbonate-hosted faunas have coexisted in Barbados from Eocene to Miocene time and we have just accidentally sampled an Eocene example of the mudstone-hosted fauna and an early Miocene example carbonate-hosted fauna for our Sr isotope work. Further stratigraphic and taxonomic work on these deposits and their faunas may solve this question and could certainly provide further insights into the evolution of the Caribbean seep fauna.

In Colombia, our attempt at Sr isotope stratigraphy failed in cases of the Sta. Clara and Mata Cana sites. In case of the Palmar-Molinera-road site, it suggests an early Miocene instead of an Oligocene age as indicated by the sample label. This early Miocene age should be treated cautiously, because the samples show evidence of diagenetic alteration. However, the similarity of *Bathymodiolus palmarensis* to the bathymodiolin found in the Miocene Freeman’s Bay and Jordan Hill seep limestones in Trinidad supports a Miocene age of the Palmar-Molinera-road site. Clearly, more detailed stratigraphic work on the Colombian seep faunas would provide more detailed insights into the evolution of the Cenozoic seep faunas of the Caribbean region. Sr isotope stratigraphy has proven useful in many, but not all, cases in the present study, another potential way forward may be dinoflagellate biostratigraphy, as recently used for Cretaceous seep carbonates found as float boulders on beaches in northern New Zealand [68].

Among the five Venezuelan sites, a bewildering array of contradicting stratigraphic ages is indicated by the labels and locality information associated with these fossils, by the personal assessment of an experienced regional geologist, and our Sr isotope stratigraphy approach. Most ages, however, seem to center around the Miocene (Fig 18).

### Biogeography & Evolution

**Fig 19:**
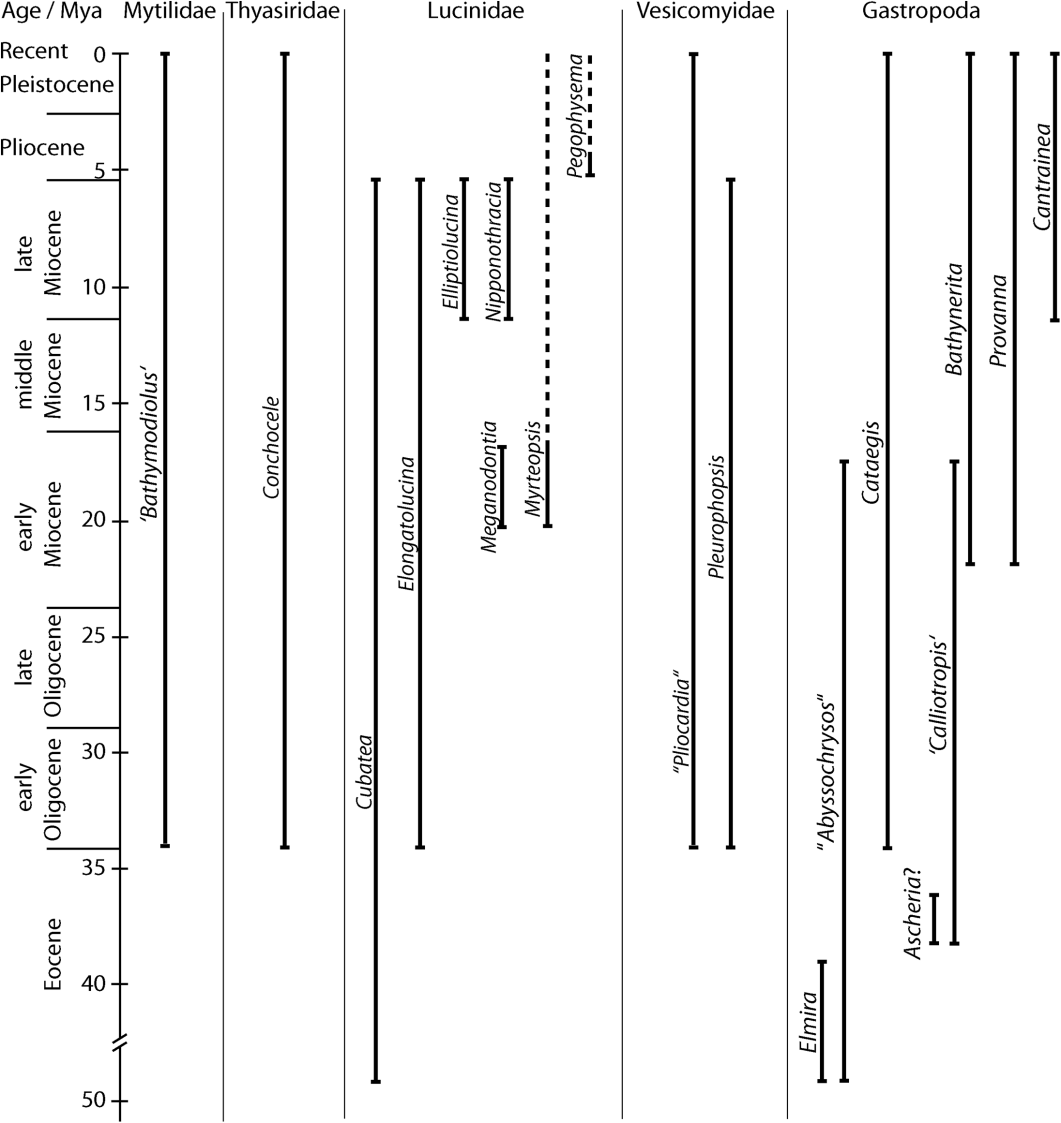
Geologic ranges of mollusk genera from Cenozoic seeps in the Caribbean region. Note break in time scale below 40 Ma; dotted lines indicate occurrences outside the seep environment; data for *Bathynerita* and ‘*Calliotropis*’ from [14], for *Ascheria* from [33].

Through the Cenozoic, seep faunas in the Caribbean and Gulf of Mexico region show three successive faunal associations:

- The Eocene faunas are dominated by lucinid bivalves and large gastropods, namely the globular *Elmira* and the tall abyssochrysoids. Similar large gastropods, as well as the lucinid bivalves, are known from various Cretaceous to Eocene seep deposits worldwide [34, 35, 43, 44, 68], and S. Kiel and T. Nobuhara, unpublished observations]. The disappearance of the large gastropods from the Caribbean seeps after the Eocene coincides with their disappearance worldwide, while the lucinid bivalve genera survived into the Oligocene and Miocene, both in the Caribbean region and in general. Thus the Eocene Caribbean seep fauna is clearly derived from, and shares a common fate with, the global seep fauna of its time.
- The Oligocene to Miocene Caribbean seep faunas consist partly of members of widely distributed lucinid genera [35], and they also saw the rise of the *Pleurophopsis*-*Cataegis*-fauna. These taxa have not been reported from other well-studied seep faunas of this age, namely in the western USA [69, 70], Japan [59, 71], and New Zealand [72]. However, this fauna does show links to the Pacific Ocean, especially during the Oligocene, when *Pleurophopsis* and other taxa reported here from Colombia are known from seep deposits in northern Peru [15, 37].
- Arguably the most remarkable faunal change among Caribbean/Gulf of Mexico seep faunas can be seen between the late Miocene and the modern fauna, when *Pleurophopsis* disappeared along with virtually all lucinid genera (Fig 19).

The latter is noteworthy because present-day Caribbean/Gulf of Mexico seeps are inhabited mostly by a distinct group of medium-sized (up to 70 mm length) lucinids which so far have no fossil record [35, 73, 74]. Although the fossil record currently lacks the resolution to narrow down the exact timing of this extinction, it roughly coincides with the closure of the Isthmus of Panama [75]. This event resulted in increasingly oligotrophic conditions in the Caribbean Sea [76, 77] and it is tempting to speculate that this had a negative effect on the large lucinids: Lucinids are only facultative chemosymbiotic and derive a significant proportion of their nutrition from suspended organic matter [78]. Thus a decrease in food availability may have led to the disappearance of the large lucinids that were used to more eutrophic conditions, and those that replaced them could not realize such large size. Indeed, at least two of the large lucinid genera that inhabited Miocene seeps in the Caribbean region (*Elliptiolucina* and *Meganodontia*) are today restricted to the more eutrophic waters of the central Indo-Westpacific [79, 80]. To the best of our knowledge, there are no studies on the size of suspension feeders on either side of the Isthmus of Panama before and after its closure, but a general decrease in the maximum size of tropical lucinid bivalves through the Cenozoic was attributed to decreasing productivity [81]. Likewise, the generally larger body size among soft-bottom feeding guilds in the western compared to the eastern North Atlantic Ocean was considered related to the higher productivity of the western North Atlantic Ocean [82].
